# Design, synthesis and antifungal activity of novel 1,4-benzoxazin-3-one derivatives containing an acylhydrazone moiety

**DOI:** 10.3389/fchem.2023.1233443

**Published:** 2023-07-20

**Authors:** Chenghao Tang, Wenbo Guo, Shengzhou Yang, Xiuhong Hu, Xingju Chen, Xiang Wang

**Affiliations:** School of Life and Health Science, Kaili University, Kaili, China

**Keywords:** 1,4-benzoxazin-3-one, acylhydrazone, phytopathogenic fungi, antifungal activity, median effective concentration

## Abstract

A series of 1,4-benzoxazin-3-one derivatives containing an acylhydrazone moiety were designed, synthesized and evaluated for their *in vitro* antifungal activities against *Gibberella zeae*, *Pellicularia sasakii*, *Phytophthora infestans*, *Capsicum wilt*, and *Phytophthora capsica*. The structures of target compounds were characterized by ^1^H NMR, ^13^H NMR, ^19^F NMR and HRMS. The preliminary antifungal evaluation of all target compounds showed that some target compounds possessed moderate to good activities against *G. zeae*, *P. sasakii*, *P. infestans* and *C. wilt*. Among them, compounds **5L** and **5o** exhibited noticeable inhibition effects against *G. zeae* with the EC_50_ values (effective concentration for 50% activity) of 20.06 and 23.17 *μ*g/ml, respectively, which were even nearly double effective than that of hymexazol (40.51 *μ*g/ml). Meanwhile, compound **5q** displayed a notable inhibitory effect toward *P. sasakii*, with the EC_50_ value of 26.66 μg/ml, which was better than that of hymexazol (32.77 *μ*g/ml). In addition, compound **5r** yielded the EC_50_ value of 15.37 *μ*g/ml against *P. infestans*, which was less than those of hymexazol (18.35 *μ*g/ml) and carbendazim (34.41 *μ*g/ml). Eventually, compound **5p** showed higher inhibitory effect against *C. wilt*, with EC_50_ value of 26.76 *μ*g/ml, which was better than that of hymexazol (>50 *μ*g/ml).

## 1 Introduction

Phytopathogenic fungi can invade plants and cause plant diseases, which not only bring about dramatic financial lose, but also can cause food safety problem because mycotoxins produced by some plant pathogenic fungi can threaten the health of humans and animals (Fisher et al., 2018). At present, one of the most efficient and immediate strategies to prevent plant diseases caused by phytopathogenic fungi is to use chemical fungicides. Meanwhile, the serious pesticide resistance, pesticide interaction and environmental pollution had been dramatically increased with the use of long-term and frequently traditional chemical fungicides (Yang et al., 2020). Therefore, there is a pressing need for the development of green fungicides with a new type of molecular scaffolds or new action mechanisms appears urgent necessary.

**FIGURE 1:**
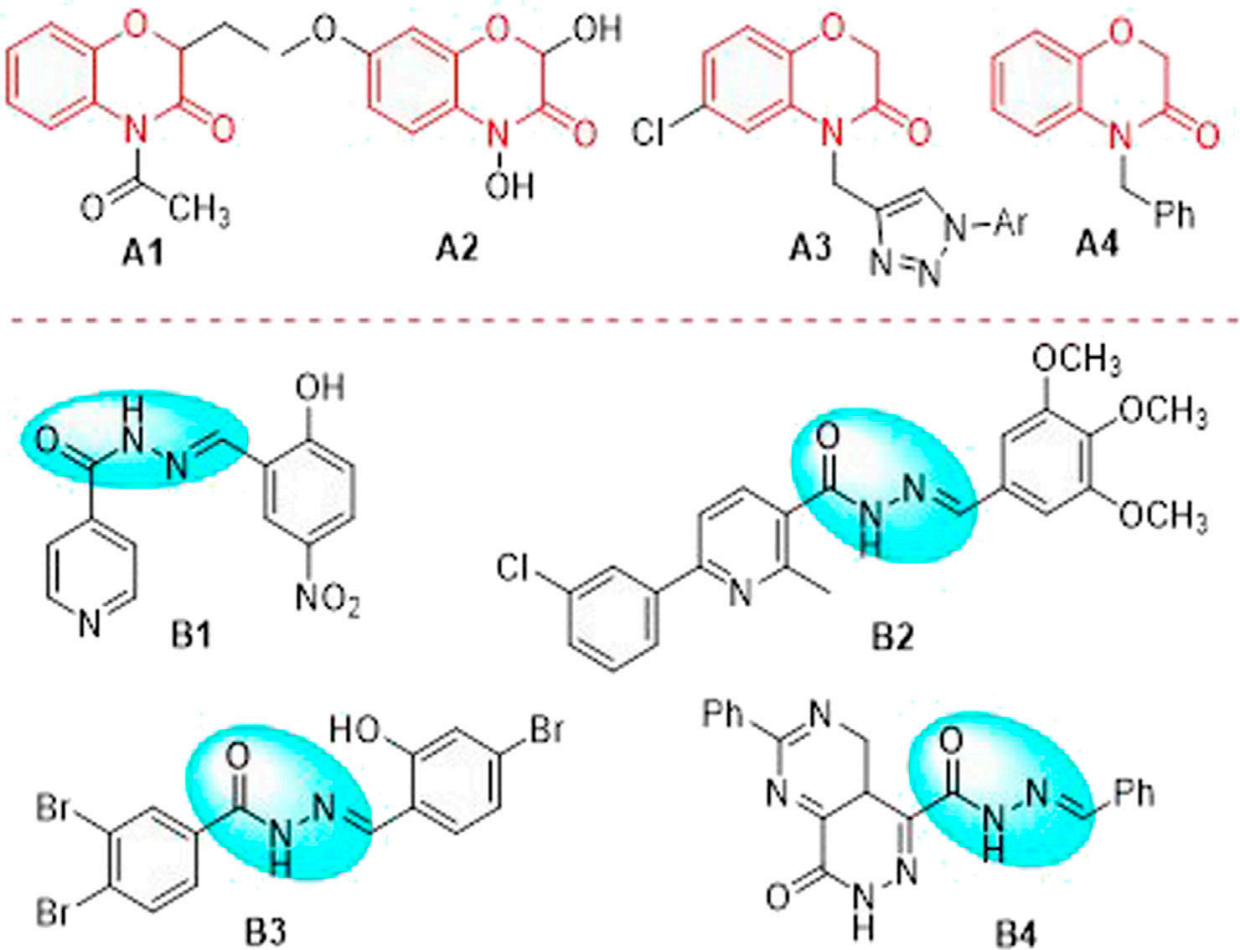
Some 1,4-benzoxazin-3-one derivatives and acylhydrazone derivatives selected with good antifungal activities.

1,4-Benzoxazin-3-one, firstly reported in rye in the 1960 s (Hietala et al., 1960), possesses a pivotal scaffold structure in a large amount of nature products and pharmaceutical molecules as well as some useful building fragments in organic synthetic chemistry (Ylijoki and Kündig, 2011). Thus, 1,4-benzoxazin-3-one derivatives have attracted many interesting attentions of chemists and pharmacologist, which proved that the introduction of this bioactive framework may give rise to high potential biological activities such as herbicidal (Wang et al., 2021), antitumor (Yang T. et al., 2019), anticonvulsant (Piao et al., 2008), antioxidants (Sonia et al., 2014) and antibacterial (Konda et al., 2015) properties, and so on. Quinolone analogue is one of the classical antimicrobial agents containing the 1,4-benzoxazine ring in its structure. Recently, this pharmacophore core has been introduced in antifungal agents (Figure 1). For example, compound **A1** is efficiently synthesized and strongly inhibited the growth of many different strains of phytopathogenic fungi (for example, *F. culmorum, R. solani, P. betae, P. cactorum, B. cinerea* and *F. oxysporum*) (Śmist et al., 2016). Compound **A2** has been found good inhibitory activities against some strains of reported fungi, such as *Saccharomyces cerevisiae* (Gleńsk et al., 2016). Compound **A3** possesses strong inhibitory activities against *Candida albicans* MTCC 3017, *Candida albicans* ATCC 90028 and *Candida glabrata* ATCC 90030 (Bollu et al., 2017). Compound **A4** exhibits promising antifungal activities toward a wide spectrum of fungi, especially the synthetic compounds with benzyl groups on the nitrogen atom exhibited prominent antifungal activity (Zamani et al., 2021). Thus, the development of efficient and convenient synthetic strategy for bioactive backbone of 1,4-benzoxazin-3-one derived antifungal agents is of great interest.

Acylhydrazone (-CO-NH-N = CH-) core can be obtained by the condensation of a hydrazide with a ketone or an aldehyde, which is a particular type of Schiff base compound. As a result, acylhydrazone core has become one of the most extensively existed pharmacophores during the course of new drug research and creation due to their privileged structures and a large number of excellent bioactive properties, such as anticancer (Vilková et al., 2022), antiviral (Yang Z. et al., 2019), antibacterial (Zhou et al., 2018), anti-inflammatory (Hernández et al., 2012), antimalarial (Shaikh et al., 2021) and insecticidal (Ren et al., 2021) properties. Recently, it is worthy of attraction that some acylhydrazone derivatives have been highlighted as potential antifungal agents (Figure 1). For instance, compound **B1** has been proved that it possesses potential antifungal activity against multiple *Candida* spp. (Backes et al., 2015). Compound **B2** has displayed excellent activities against some fungi, such as *Aspergillus fumigatus* and *Candida albicans*, with minimum inhibitory concentration of 0.98 and 0.49 *μ*g/ml, respectively, which is better than that of amphotericin B (Guilherme et al., 2019). Compound **B3** has been demonstrated that it displayed a wide spectrum of antifungal activities against many clinically relevant fungal strains, such as *C. albicans*, *C. krusei*, *C. krusei*, *C. parapsilosis, and A. fumigatus* (Haranahalli et al., 2019)*.* Compound **B4** has exhibited excellent inhibition against *P. brasiliensis* and *Candida* spp. with minimum inhibitory concentration of 0.5 *μ*g/ml (Rozada et al., 2020).

**FIGURE 2:**
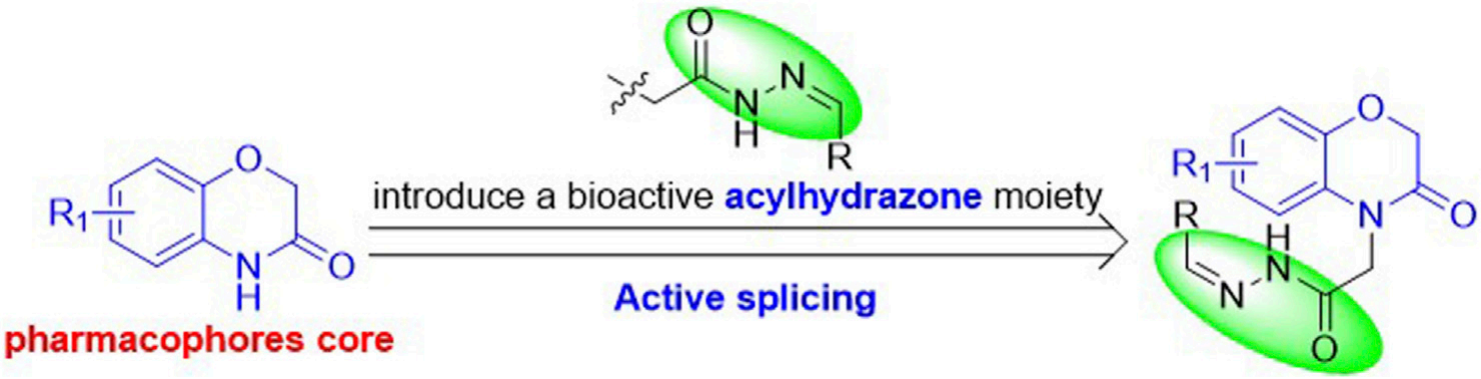
The design of the title compounds.

Thus, to improve the antifungal activities of 1,4-benzoxazin-3-one derivatives, we committed to introducing the bioactive acylhydrazone moiety to 1,4-benzoxazin-3-one skeleton, then, designing and synthesizing a serial of novel 1,4-benzoxazin-3-one derivatives with an active acylhydrazone moiety (Figure 2) and appraised for their *in vitro* antifungal activities against *Gibberella zeae* (*G. zeae*), *Pellicularia sasakii* (*P. sasakii*), *Phytophthora infestans* (*P. infestans*), *Capsicum wilt* (*C. wilt*) and *Phytophthora capsica* (*P. capsica*).

## 2 Experimental

### 2.1 Chemicals and reagents

The methodology for the synthesis of the title compounds **5a**–**5s** was presented in Scheme 1. The title compound **5** was obtained by the cyclization, substitution, hydrazinolysis and condensation reactions with 36%–53% yields over four steps. The structures of the title compound **5** were confirmed by ^1^H NMR, ^13^C NMR, ^19^F NMR and HRMS, and all analytical data were consistent with the assigned structures. The Supplementary Material contain the ^1^H NMR, ^13^C NMR, ^19^F NMR and HRMS spectra of the target compound **5** (Supplementary Figures S1–S66).

**SCHEME 1:**
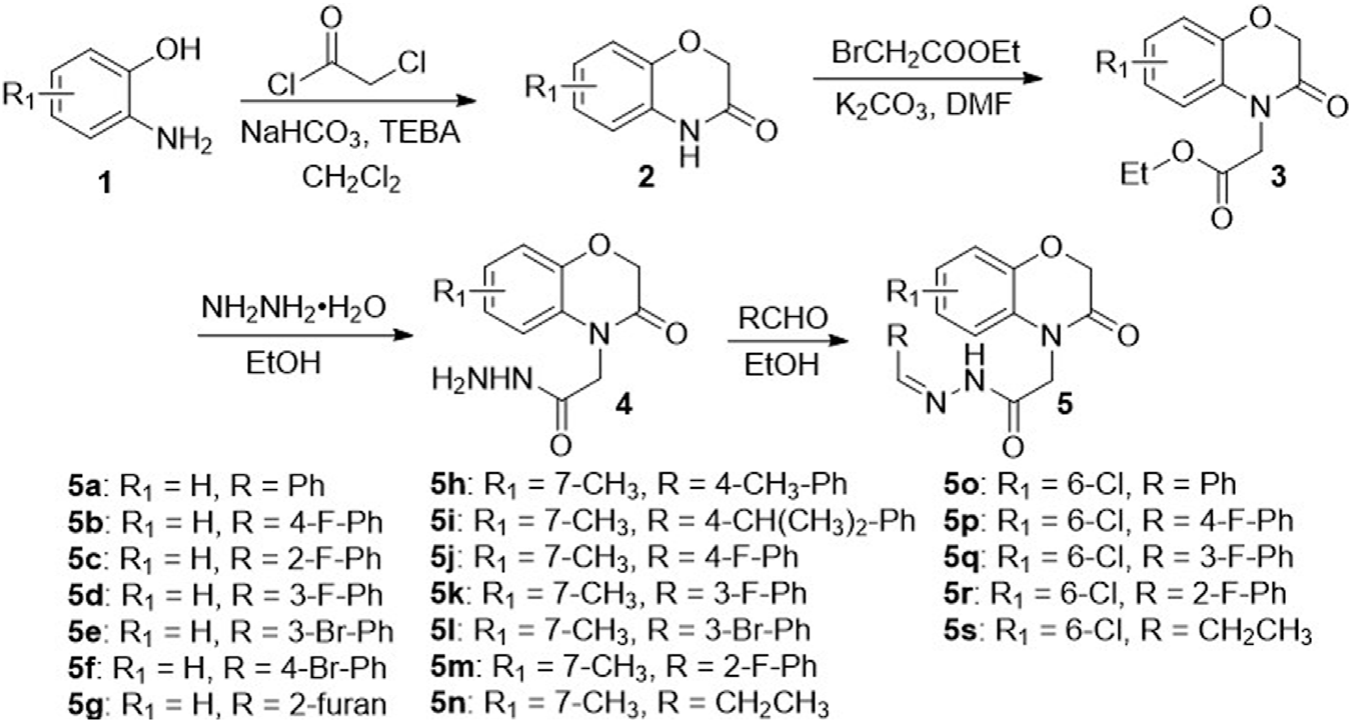
Synthetic route of the target compounds **5a**–**5s.**

### 2.2 Synthesis of intermediates 2–4

*o*-Amino phenol (3 g, 30 mmol), benzyl triethyl ammonium chloride (TEBA, 6.27 g, 30 mmol) and dichloromethane (20 ml) was added to a 100 ml reaction flask equipped with a magnetic stir bar. After the mixture was stirred for 10 min, sodium hydrogen carbonate (9.18 g, 108 mmol) was added step into mixture. The resultant mixture was allowed to stir at 0°C, and chloroacetyl chloride (3.66 g, 32.4 mmol) was added *via* syringe over a period of 15 min. Then, the reactant mixture was refluxed at 40°C for 6–8 h. After the reactants were consumed (TLC analysis), the resulting crude residue was poured into ice water (20 ml). The crude products were collected by filtration, washed with water, and recrystallized from methanol to obtain intermediate **2** (Benarjee et al., 2022).

The intermediate **2** (1.49 g, 10 mmol) and K_2_CO_3_ (1.38 g, 10 mmol) were added into a 100 ml reaction flask with 20 ml dimethylformamide (DMF). Then, ethyl bromoacetate (1.84 g, 11 mmol) was dropwise added via syringe and the reaction mixture was allowed to stir at rt for 12 h. After the reactants were consumed (TLC analysis), the resulting crude residue was extracted with ethyl acetate (3 × 20 ml). The organic layer was washed with water, dried over anhydrous Na_2_SO_4_, concentrated under reduced pressure to obtain intermediate **3** (Safakish et al., 2020).

Intermediate **3** was dissolved in 10 ml ethanol and heated at around 60°C about 10 min. Then, hydrazine hydrate (4 equiv.) was dropwise added via syringe to this mixture. The reaction mixture was refluxed for 6–8 h, and then allowed to pour into ice water. The crude residue was filtered to yield the intermediate **4** (Zhang et al., 2022).

### 2.3 Synthesis of the title compound 5

Intermediate **4** was dissolved in 10 ml ethanol, and the solution was gently heated at around 60°C. Then, corresponding substituent aldehyde (1 equiv.) in 5 ml ethanol was dropwise added via syringe into this solution. The reaction mixture was refluxed for 2–4 h, and allowed to cool to room temperature. The crude residue was filtered and recrystallized from toluene to get the title compound **5**.

## 3 Results and discussion

### 3.1 Chemistry

The nuclear magnetic resonance (NMR) spectroscopy is a practical strategy to identify the *cis-trans* isomers in acylhydrazones (Lopes et al., 2013). The analysis of the ^1^H NMR spectra displayed that title compounds **5a**–**5s** exist as *Z*/*E* isomers, and the ratio of *Z*/*E* isomers can be confirmed *via* ^1^H NMR based on the previously reported literature method (Palla, 1986). The chemical shift of ^1^H NMR spectrum of the title compounds **5a**–**5s** show slightly narrow singlets for *N–H* group at 11.26–11.84 ppm. The characteristic singlets of imine hydrogens appear between 7.38 and 8.46 ppm with the *cis-trans* isomers, which control the *Z*/*E* isomers ratio of the title compound **5** synthesized from our approach is 3:1 according to previously reported literatures (Munir et al., 2021). The methylene moiety of 1,4-benzoxazin-3-one ring appeared at around 4.66 and 5.05 ppm also presents 3:1 *Z*/*E* isomers ratio, which is consistent with the *Z*/*E* isomers ratio of imine hydrogens. ^13^C NMR exhibited peaks at around 165.0 and 168.0 ppm for carbonyl groups of *N*-acylhydrazone and 1,4-benzoxazin-3-one ring portion. The signal of imine carbon is meant to appear at 142.8–145.1 ppm. For example, the signal of imine hydrogens of the title compound **5a** appears at 8.06 and 8.24 ppm, with the *Z*/*E* isomers ratio of 3:1. The chemical shift of methylene moiety of 1,4-benzoxazin-3-one ring appeared at 5.08 and 4.67 ppm, also with the *Z*/*E* isomers ratio of 3:1. Finally, the ESI-HRMS spectrum revealed an obvious signal at 332.1006, which was assigned to the [M + Na]^+^ species of the target compound **5a**.

### 3.2 Antifungal activity

All synthesized 1,4-benzoxazin-3-one derivatives in this work were screened for *in vitro* fungicidal activities against five types of plant pathogenic fungi including *G. zeae*, *P. sasakii*, *P. infestans*, *C. wilt* and *P. capsica* at the concentration of 50 *μ*g/ml through the classical mycelium growth rate method according to the previously established procedure (see Supplementary Materials for full procedure details)*.* Commercial agrochemicals hymexazol and carbendazim were used as positive fungicides against fungal strains. The obtained results are depicted in Table 1. The results of preliminary screening indicated that some of synthesized 1,4-benzoxazin-3-one derivatives also had moderate to good activities to inhibit the growth of *G. zeae*, *P. sasakii*, *P. infestans* and *C. wilt* fungal strains, but almost all the title compounds failed to exhibit effective inhibition effects against *P. capsica* in this work*.* Firstly, for *G. zeae*, compounds **5L** and **5o** exhibited excellent bioactive with the inhibitory rates 76.37% and 76.14%, respectively, which were superior to hymexazol (49.47%). Secondly, compounds **5L** and **5q** displayed stronger inhibitory activities against *P. sasakii*, with the inhibition rates of 64.38% and 73.32%, respectively, than that of hymexazol (60.70%). Meanwhile, compound **5r** exhibited the excellent inhibitory activity against *P. infestans*, with the inhibition rate of 82.62%, which was better than hymexazol (72.86%) and carbendazim (58.57%). In addition, compound **5p** had 71.33% inhibition rate against *C. wilt*, which was better than hymexazol (49.88%).

**TABLE 1:** *In vitro* fungicidal activity of the title compound **5** against tested fungi.

Comp	Mycelium growth inhibitory rate (%) at 50 *μ*g/ml				
	*G. zeae*	*P. sasakii*	*P. infestans*	*C. wilt*	*P. capsici*
**5a**	36.84 ± 5.75	43.44 ± 2.49	44.76 ± 3.58	46.39 ± 1.91	24.25 ± 1.68
**5b**	13.22 ± 2.07	44.89 ± 2.31	34.76 ± 3.58	54.78 ± 2.28	15.41 ± 1.28
**5c**	24.68 ± 1.92	44.16 ± 2.69	33.10 ± 4.56	43.59 ± 1.44	17.56 ± 1.50
**5d**	20.01 ± 3.07	45.13 ± 3.03	47.86 ± 1.97	24.01 ± 1.91	25.45 ± 2.03
**5e**	19.77 ± 1.64	42.24 ± 2.58	61.90 ± 1.73	19.11 ± 1.38	23.30 ± 2.68
**5f**	19.30 ± 2.91	43.92 ± 1.69	33.81 ± 1.73	22.14 ± 1.02	32.38 ± 1.68
**5g**	35.91 ± 4.13	45.37 ± 3.35	37.38 ± 2.29	25.64 ± 2.24	29.51 ± 2.11
**5h**	31.23 ± 3.32	41.28 ± 2.98	55.48 ± 3.55	45.69 ± 2.41	27.60 ± 1.50
**5i**	30.99 ± 3.00	39.83 ± 1.75	43.10 ± 3.31	41.03 ± 2.06	31.42 ± 1.68
**5j**	50.18 ± 4.92	50.66 ± 1.09	41.19 ± 1.08	52.91 ± 1.69	34.05 ± 2.03
**5k**	30.29 ± 4.13	46.33 ± 1.42	37.14 ± 2.02	40.09 ± 2.57	27.36 ± 2.51
**5l**	**76.37 ± 2.07**	**64.38 ± 4.35**	**38.33 ± 2.77**	**43.59 ± 2.45**	**18.28 ± 2.03**
**5m**	37.78 ± 4.04	43.20 ± 4.15	32.14 ± 2.35	38.23 ± 1.05	16.13 ± 1.50
**5n**	37.31 ± 4.50	44.89 ± 3.47	39.52 ± 4.30	41.96 ± 2.30	30.47 ± 1.50
**5o**	**76.14 ± 1.26**	**54.27 ± 1.75**	**32.14 ± 1.97**	**40.33 ± 1.44**	**33.33 ± 1.50**
**5p**	**33.10 ± 6.86**	**44.40 ± 3.26**	**33.57 ± 2.17**	**71.33 ± 1.47**	**32.86 ± 2.11**
**5q**	**51.11 ± 3.81**	**73.32 ± 1.09**	**43.10 ± 1.40**	**39.16 ± 1.47**	**32.14 ± 1.48**
**5r**	**39.18 ± 3.03**	**44.16 ± 4.63**	**82.62 ± 1.08**	**48.02 ± 2.06**	**15.17 ± 2.11**
**5s**	34.50 ± 2.29	38.15 ± 3.81	34.29 ± 2.02	43.59 ± 1.44	13.74 ± 1.68
Hymexazol	49.47 ± 1.26	60.77 ± 1.69	72.86 ± 2.02	49.88 ± 1.05	27.60 ± 1.50
Carbendazim	94.85 ± 1.70	81.95 ± 1.51	58.57 ± 1.28	68.30 ± 1.44	33.81 ± 2.11

The compounds in bold showed better antifungal activities than other compounds.

To further validate the antifungal activities of the title compounds, the EC_50_ values of some title compounds against *G. zeae*, *P. sasakii*, *P. infestans*, and *C. wilt* were tested and the result data were demonstrated in Table 2. Our results indicated that compounds **5L** and **5o** exhibited commendable *in vitro* antifungal activities against *G. zeae*, with the EC_50_ values of 20.06, and 23.17 *μ*g/ml, respectively, which are nearly double effective than that of hymexazol (40.51 *μ*g/ml). Meanwhile, compound **5q** displayed outstanding inhibitory activity against *P. sasakii*, with the EC_50_ value of 26.66 *μ*g/ml, respectively, which is better than hymexazol (32.77 *μ*g/ml). Furthermore, compound **5r** also had excellent inhibitory activity against *P. infestans*, with EC_50_ value of 15.37 *μ*g/ml, which is double more efficient compared to that of carbendazim (34.41 *μ*g/ml). Then, the EC_50_ values of compounds **5b** and **5p** against to *C. wilt* were calculated, just the EC_50_ of **5p** (26.76 *μ*g/ml) was slightly close to that of carbendazim (26.08 *μ*g/ml).

**TABLE 2:** The EC_50_ values of selected target compounds against tested fungi.

Pathogens	Comp	Regression equation	EC_50_ (*u*g/mL)	Pathogens	Comp	Regression equation	EC_50_ (*u*g/mL)
*G. zeae*	**5L**	y = 1.7566x + 2.7123	20.06 ± 0.26	*P. infestans*	**5e**	y = 1.3509x + 3.0713	26.77 ± 0.39
	**5o**	y = 2.0930x + 2.1434	23.17 ± 0.13		**5r**	y = 1.8439x + 2.8121	15.37 ± 0.07
	Hymexazol	y = 1.6843x + 2.2924	40.51 ± 1.85		Hymexazol	y = 1.4933x + 3.1129	18.35 ± 0.51
	Carbendazim	y = 1.7550x + 3.4522	7.62 ± 0.02		Carbendazim	y = 1.6463x + 2.4701	34.41 ± 2.43
*P. sasakii*	**5L**	y = 2.0253x + 1.9925	30.55 ± 1.25	*C. wilt*	**5b**	y = 1.7469x + 1.8574	>50
	**5q**	y = 1.9901x + 2.1623	26.66 ± 0.36		**5p**	y = 1.5894x + 2.7575	26.76 ± 0.57
	Hymexazol	y = 2.0632x + 1.8785	32.77 ± 1.47		Hymexazol	y = 1.7360x + 1.6936	>50
	Carbendazim	y = 1.8675x + 2.7220	16.59 ± 0.42		Carbendazim	y = 1.7876x + 2.4682	26.08 ± 1.25

The compounds in bold showed better antifungal activities than other compounds.

For compounds **5a**-**5g** (no substituents attached on 1,4-benzoxazin-3-one skeleton), only compound **5e** showed better activity against *P. infestans*, with 3-Br attached aryl of acylhydrazone, the introduction of other substituents, including 4-F-Ph, 2-F-Ph, 3-F-Ph, 4-Br-Ph and 2-furan could not obviously affect the antifungal activities. Then, some of target compounds showed better activity against fungal strains, when introduction of methyl attached on 1,4-benzoxazin-3-one skeleton (for compounds **5h**-**5n**), for example, compound **5L** (3-Br attached aryl of acylhydrazone) exhibited bioactive with the inhibitory rates 76.37% and 64.38% against *G. zeae* and *P. sasakii*, respectively. However, target compounds (**5o** – **5t**), with 6-Cl attached on 1,4-benzoxazin-3-one skeleton, exhibited better antifungal activity than other substituents attached on 1,4-benzoxazin-3-one skeleton, such as compound **5o**, **5p**, **5q** and **5s** were exhibited excellent bioactive with the inhibitory rates 76.14%, 71.33%, 73.32% and 82.62% against *G. zeae*, *C. wilt*, *P. sasakii* and *P. infestans*, with EC_50_ value of 23.17, 26.76, 26.66 and 15.37 *μ*g/ml, respectively. Especially, compound **5s** had the best activity against *P. infestans* fungal strain. The replacement of the benzene ring (**5o**) to alkyl such as ethyl (**5t**) group could not improve the antifungal activity.

## 4 Conclusion

In conclusion, a total of 19 original 1,4-benzoxazin-3-one derived compounds containing an acylhydrazone moiety were synthesized and estimated for their *in vitro* antifungal activities against five species of fungi. The preliminary antifungal evaluation of all the title compounds indicated that some of them had considerable activities against tested fungi. Among them, compounds **5L** and **5o** possessed commendable outstanding antifungal activity toward *G. zeae* with EC_50_ values of 20.06 and 23.17 *μ*g/ml, respectively. Meanwhile, compound **5q** showed outstanding inhibitory activity against *P. sasakii,* with EC_50_ value of 26.66 *μ*g/ml. Additionally, compounds **5e** and **5r** had preeminent inhibitory activity against *P. infestans,* with EC_50_ values of 26.77 and 15.37 *μ*g/ml, respectively. Our present work indicated that 1,4-benzoxazin-3-one derivatives with an acylhydrazone moiety could result in potential fungicide agents.

## Data Availability

The original contributions presented in the study are included in the article/Supplementary Material, further inquiries can be directed to the corresponding author.
