# Supplementary material for: Design, synthesis and antifungal activity of novel 1,4-benzoxazin-3-one derivatives containing an acylhydrazone moiety
Source: Front Chem. 2023 Jul 20;11:1233443. doi: 10.3389/fchem.2023.1233443 (PMC10400319; doi:10.3389/fchem.2023.1233443)
Supplement: Supplementary file 1 [file DataSheet1.doc]

***Supplementary Material***

**Design, Synthesis and Antifungal Activities of Novel 1,4-Benzoxazin-3-one Derivatives Containing an Acylhydrazone Moiety**

**Chenghao Tang^*^, Wenbo Guo, Shengzhou Yang, Xiuhong Hu, Xingju Chen, Xiang Wang**

School of Life and Health Science, Kaili University, Kaili, China

*Corresponding author. E-mail: [chtang1122@163.com](mailto:chtang1122@163.com) (Chenghao Tang)

**Contents**

Procedure for antifungal assay of the title compounds S2

Characterization of substrates and products S2

^1^H NMR, ^13^C NMR and ^19^F NMR spectra and HRMS of products S9

Table S1 *In vitro* fungicidal activity of target compounds against tested fungi S48

Table S2 The EC_50_ values of selected target compounds against tested fungi S48

**Procedure for antifungal assay of the title compounds**

The in vitro antifungal effects of the title compounds against *Gibberella zeae*, *Pellicularia sasakii*, *Phytophthora infestans*, *Capsicum wilt*, and *Phytophthora capsica* were evaluated using a mycelium growth rate method that is briefly described as follows: 100 *μ*L dimethylsulfoxide (DMSO) dissolved in a tested compound was added into 45 mL of potato dextrose agar (PDA). After shaking well, the obtained mixture was equally divided and poured into three ninecentimeter Petri plates. Equal DMSO and the commercial agricultural fungicides hymexazol and carbendazim were used as the blank and positive controls, respectively. Then, a mycelia dish with a diameter of 5 mm was aseptically inoculated in the center of the above PDA plate with three replicates. The inoculated plates were incubated at 25 ± 1 °C for 3−7 days in a dark environment. After the mycelium diameter of the blank control reached 7.0−7.5 cm, the radial growth of the fungal colonies was measured and the data were statistically analyzed. The title compounds, which had inhibitions that exceeded 50% at 10 *μ*g/mL, were further tested for their antifungal effects at five double-declining concentrations to calculate the corresponding EC_50_ values using SPSS 11.5 software.

**Characterization of substrates and products**

*(Z)-N'-benzylidene-2-(3-oxo-2,3-dihydro-4H-benzo[b][1,4]oxazin-4-yl)acetohydrazide (****5a****)*

White solid; 45% yield over 4 steps, m.p. 245-247 ^o^C; *Z*/*E* = 3:1, ^1^H NMR (400 MHz, DMSO-*d_6_*) δ 11.73 (s, 1H, -*NH*), 8.24 (for *E* isomer, s, 0.25H, CONHN−*CH*), 8.06 (for *Z* isomer, s, 0.76H, CONHN−*CH*), 7.81 – 7.65 (m, 2H), 7.45 (dt, *J* = 6.0, 1.7 Hz, 3H), 7.11 – 6.92 (m, 4H), 5.08 (for *Z* isomer, s, 1.50H), 4.72 (d, *J* = 4.3 Hz, 2H), 4.67 (for *E* isomer, s, 0.48H); ^13^C NMR (101 MHz, DMSO-*d_6_*) δ 168.4, 165.1, 165.0, 163.8, 147.6, 145.1, 144.5, 134.5, 130.5, 129.6, 129.3, 127.6, 127.5, 124.0, 123.2, 117.0, 116.0, 67.4, 42.8; HRMS (ESI, m/z): Mass calcd. for C_17_H_15_N_3_O_3_ [M+Na]^+^ 332.1006, found 332.1006.

*(Z)-N'-(4-fluorobenzylidene)-2-(3-oxo-2,3-dihydro-4H-benzo[b][1,4]oxazin-4-yl)acetohydrazide (****5b****)*

White solid; 51% yield over 4 steps, m.p. 248-250 ^o^C; *Z*/*E* = 3:1, ^1^H NMR (400 MHz, DMSO-*d_6_*) δ 11.74 (s, 1H, -*NH*), 8.24 (for *E* isomer, s, 0.25H, CONHN−*CH*), 8.05 (for *Z* isomer, s, 0.75H, CONHN−*CH*), 7.85 – 7.72 (m, 2H), 7.29 (t, *J* = 8.9, Hz, 2H), 7.08 – 6.97 (m, 4H), 5.08 (for *Z* isomer, s, 1.47H), 4.72 (d, *J* = 4.2 Hz, 2H), 4.67 (for *E* isomer, s, 0.49H); ^13^C NMR (101 MHz, DMSO-*d_6_*) δ 168.4, 165.1, 163.8, 162.3, 145.1, 143.4, 131.1, 129.7, 129.6, 124.0, 123.2, 117.0, 116.5, 116.3, 116.2, 116.0, 115.9, 67.4, 42.8; ^19^F NMR (376 MHz, DMSO) δ -110.44, -110.65; HRMS (ESI, m/z): Mass calcd. for C_17_H_14_FN_3_O_3_ [M+Na]^+^ 350.0911, found 350.0908.

*(Z)-N'-(2-fluorobenzylidene)-2-(3-oxo-2,3-dihydro-4H-benzo[b][1,4]oxazin-4-yl)acetohydrazide (****5c****)*

White solid; 46% yield over 4 steps, m.p. 233-235 ^o^C; *Z*/*E* = 3:1, ^1^H NMR (400 MHz, DMSO-*d_6_*) δ 11.73 (s, 1H, -*NH*), 8.46 (for *E* isomer, s, 0.25H, CONHN−*CH*), 8.26 (for *Z* isomer, s, 0.76H, CONHN−*CH*), 8.04 – 7.83 (m, 1H), 7.55 – 7.44 (m, 1H), 7.36 – 7.24 (m, 2H), 7.08 – 6.97 (m, 4H), 5.09 (for *Z* isomer, s, 1.53H), 4.72 (d, *J* = 4.3 Hz, 2H), 4.67 (for *E* isomer, s, 0.49H); ^13^C NMR (101 MHz, DMSO-*d_6_*) δ 168.5, 165.1, 163.9, 162.4, 145.1, 137.4, 137.3, 132.5, 132.4, 129.5, 127.0, 127.0, 125.4, 124.2, 124.0, 123.2, 122.1, 122.0, 117.0, 116.6, 116.4, 116.0, 115.9, 67.4, 42.8; ^19^F NMR (377 MHz, DMSO) δ -121.02, -121.17; HRMS (ESI, m/z): Mass calcd. for C_17_H_14_FN_3_O_3_ [M+Na]^+^ 350.0911, found 350.0908.

*(Z)-N'-(3-fluorobenzylidene)-2-(3-oxo-2,3-dihydro-4H-benzo[b][1,4]oxazin-4-yl)acetohydrazide (****5d****)*

White solid; 39% yield over 4 steps, m.p. 242-244 ^o^C; *Z*/*E* = 3:1, ^1^H NMR (400 MHz, DMSO-*d_6_*) δ 11.84 (s, 1H, -*NH*), 8.25 (for *E* isomer, s, 0.23H, CONHN−*CH*), 8.05 (for *Z* isomer, s, 0.77H, CONHN−*CH*), 7.65 – 7.48 (m, 3H), 7.32 – 7.22 (m, 1H), 7.06 – 6.99 (m, 4H), 5.10 (for *Z* isomer, s, 1.44H), 4.72 (d, *J* = 4.1 Hz, 2H), 4.69 (for *E* isomer, s, 0.55H); ^13^C NMR (101 MHz, DMSO-*d_6_*) δ 168.6, 165.1, 165.0, 164.1, 164.0, 161.7, 145.1, 143.1, 137.1, 137.0, 131.4, 131.3, 129.5, 124.2, 124.1, 124.0, 123.2, 117.3, 117.1, 117.0, 116.0, 115.9, 113.4, 113.2, 67.4, 42.9; ^19^F NMR (377 MHz, DMSO) δ -112.79; HRMS (ESI, m/z): Mass calcd. for C_17_H_14_FN_3_O_3_ [M+Na]^+^ 350.0911, found 350.0915.

*(Z)-N'-(3-bromobenzylidene)-2-(3-oxo-2,3-dihydro-4H-benzo[b][1,4]oxazin-4-yl)acetohydrazide (****5e****)*

White solid; 46% yield over 4 steps, m.p. 261-263 ^o^C; *Z*/*E* = 3:1, ^1^H NMR (400 MHz, DMSO-*d_6_*) δ 11.73 (s, 1H, -*NH*), 8.21 (for *E* isomer, s, 0.25H, CONHN−*CH*), 8.02 (for *Z* isomer, s, 0.76H, CONHN−*CH*), 7.73 – 7.63 (m, 4H), 7.05 – 6.99 (m, 4H), 5.08 (for *Z* isomer, s, 1.53H), 4.71 (d, *J* = 4.3 Hz, 2H), 4.67 (for *E* isomer, s, 0.52H); ^13^C NMR (101 MHz, DMSO-*d_6_*) δ 168.4, 165.1, 165.0, 163.8, 147.6, 145.1, 144.5, 134.5, 130.5, 129.6, 129.3, 127.6, 127.5, 124.0, 123.2, 117.0, 116.0, 67.4, 42.8; HRMS (ESI, m/z): Mass calcd. for C_17_H_14_BrN_3_O_3_ [M+Na]^+^ 410.0111, found 410.0111.

*(Z)-N'-(4-bromobenzylidene)-2-(3-oxo-2,3-dihydro-4H-benzo[b][1,4]oxazin-4-yl)acetohydrazide (****5f****)*

White solid; 44% yield over 4 steps, m.p. 278-279 ^o^C; *Z*/*E* = 3:1, ^1^H NMR (400 MHz, DMSO-*d_6_*) δ 11.80 (s, 1H, -*NH*), 8.21 (for *E* isomer, s, 0.25H, CONHN−*CH*), 8.02 (for *Z* isomer, s, 0.76H, CONHN−*CH*), 7.73 – 7.63 (m, 4H), 7.06 – 6.98 (m, 4H), 5.08 (for *Z* isomer, s, 1.48H), 4.72 (d, *J* = 4.3 Hz, 2H), 4.67 (for *E* isomer, s, 0.47H); ^13^C NMR (101 MHz, DMSO-*d_6_*) δ 168.5, 165.1, 145.1, 143.3, 133.8, 132.3, 129.6, 129.5, 129.4, 124.0, 123.7, 123.2, 117.0, 116.0, 67.4, 42.8; HRMS (ESI, m/z): Mass calcd. for C_17_H_14_BrN_3_O_3_ [M+Na]^+^ 410.0111, found 410.0105.

*(Z)-N'-(furan-2-ylmethylene)-2-(3-oxo-2,3-dihydro-4H-benzo[b][1,4]oxazin-4-yl)acetohydrazide (****5g****)*

White solid; 40% yield over 4 steps, m.p. 207-208 ^o^C; *Z*/*E* = 3:1, ^1^H NMR (400 MHz, DMSO-*d_6_*) δ 11.68 (s, 1H, -*NH*), 8.12 (for *E* isomer, s, 0.27H, CONHN−*CH*), 7.94 (for *Z* isomer, s, 0.72H, CONHN−*CH*), 7.85 (s, 1H), 7.02 (dd, *J* = 10.3, 5.0 Hz, 4H), 6.94 (dd, *J* = 7.5, 3.4 Hz, 1H), 6.64 (ddd, *J* = 5.4, 3.5, 1.8 Hz, 1H), 5.00 (for *Z* isomer, s, 1.44H), 4.72 (d, *J* = 6.6 Hz, 2H), 4.66 (for *E* isomer, s, 0.56H); ^13^C NMR (101 MHz, DMSO-*d_6_*) δ 168.2, 165.1, 165.0, 163.7, 149.6, 149.5, 145.8, 145.6, 145.1, 145.0, 137.5, 134.7, 129.6, 129.5, 124.2, 123.9, 123.2, 117.0, 116.9, 115.9, 114.4, 114.3, 112.7, 67.4, 42.6; HRMS (ESI, m/z): Mass calcd. for C_15_H_13_N_3_O_4_ [M+Na]^+^ 322.0798, found 322.0794.

*(Z)-2-(7-methyl-3-oxo-2,3-dihydro-4H-benzo[b][1,4]oxazin-4-yl)-N'-(4-methylbenzylidene)acetohydrazide (****5h****)*

White solid; 51% yield over 4 steps, m.p. 273-274 ^o^C; *Z*/*E* = 3:1, ^1^H NMR (400 MHz, DMSO-*d_6_*) δ 11.65 (s, 1H, -*NH*), 8.19 (for *E* isomer, s, 0.24H, CONHN−*CH*), 8.01 (for *Z* isomer, s, 0.72H, CONHN−*CH*), 7.61 (dd, *J* = 14.7, 7.9 Hz, 2H), 7.26 (d, *J* = 7.9 Hz, 2H), 6.95 – 6.79 (m, 3H), 5.03 (for *Z* isomer, s, 1.50H), 4.68 (d, *J* = 5.1 Hz, 2H), 4.63 (for *E* isomer, s, 0.48H), 2.35 (s, 3H), 2.24 (s, 3H); ^13^C NMR (101 MHz, DMSO-*d_6_*) δ 168.3, 164.9, 144.9, 144.5, 140.3, 133.4, 131.8, 129.9, 127.6, 127.4, 127.1, 123.5, 117.4, 115.7, 67.5, 42.8, 21.5, 20.7; HRMS (ESI, m/z): Mass calcd. for C_19_H_19_N_3_O_3_ [M+Na]^+^ 360.1318, found 360.1317.

*(Z)-N'-(4-isopropylbenzylidene)-2-(7-methyl-3-oxo-2,3-dihydro-4H-benzo[b][1,4]oxazin-4-yl)acetohydrazide (****5i****)*

White solid; 53% yield over 4 steps, m.p. 215-217 ^o^C; *Z*/*E* = 3:1, ^1^H NMR (400 MHz, DMSO-*d_6_*) δ 11.65 (s, 1H, -*NH*), 8.19 (for *E* isomer, s, 0.25H, CONHN−*CH*), 8.01 (for *Z* isomer, s, 0.72H, CONHN−*CH*), 7.68 – 7.59 (m, 2H), 7.32 (d, *J* = 8.3 Hz, 2H), 6.97 – 6.78 (m, 3H), 5.03 (for *Z* isomer, s, 1.44H), 4.72 (d, *J* = 4.3 Hz, 2H), 4.63 (for *E* isomer, s, 0.52H), 2.99 – 2.84 (m, 1H), 2.24 (s, 3H), 1.22 (d, *J* = 6.9 Hz, 6H); ^13^C NMR (101 MHz, DMSO-*d_6_*) δ 168.3, 164.9, 151.1, 144.9, 144.5, 133.5, 132.2, 127.7, 127.5, 127.3, 127.1, 123.5, 117.4, 115.7, 67.5, 42.8, 33.8, 24.1, 20.7; HRMS (ESI, m/z): Mass calcd. for C_21_H_23_N_3_O_3_ [M+Na]^+^ 388.1632, found 388.1629.

*(Z)-N'-(4-fluorobenzylidene)-2-(7-methyl-3-oxo-2,3-dihydro-4H-benzo[b][1,4]oxazin-4-yl)acetohydrazide (****5j****)*

White solid; 43% yield over 4 steps, m.p. 248-249 ^o^C; *Z*/*E* = 3:1, ^1^H NMR (400 MHz, DMSO-*d_6_*) δ 11.72 (s, 1H, -*NH*), 8.24 (for *E* isomer, s, 0.24H, CONHN−*CH*), 8.04 (for *Z* isomer, s, 0.73H, CONHN−*CH*), 7.85 – 7.74 (m, 2H), 7.29 (t, *J* = 8.8 Hz, 2H), 6.94 – 6.79 (m, 3H), 5.05 (for *Z* isomer, s, 1.46H), 4.72 (d, *J* = 4.3 Hz, 2H), 4.64 (for *E* isomer, s, 0.51H), 2.24 (s, 3H); ^13^C NMR (101 MHz, DMSO-*d_6_*) δ 168.4, 164.9, 164.8, 163.8, 162.3, 146.5, 144.9, 143.3, 133.7, 133.5, 131.1, 129.8, 129.7, 129.6, 129.4, 128.7, 127.1, 127.0, 125.8, 123.5, 117.4, 116.5, 116.3, 115.7, 115.6, 67.5, 42.8, 20.7; ^19^F NMR (377 MHz, DMSO) δ -110.45, -110.66; HRMS (ESI, m/z): Mass calcd. for C_18_H_16_FN_3_O_3_ [M+Na]^+^ 364.1068, found 364.1063.

*(Z)-N'-(3-fluorobenzylidene)-2-(7-methyl-3-oxo-2,3-dihydro-4H-benzo[b][1,4]oxazin-4-yl)acetohydrazide (****5k****)*

White solid; 40% yield over 4 steps, m.p. 258-259 ^o^C; *Z*/*E* = 3:1, ^1^H NMR (400 MHz, DMSO-*d_6_*) δ 11.82 (s, 1H, -*NH*), 8.24 (for *E* isomer, s, 0.22H, CONHN−*CH*), 8.04 (for *Z* isomer, s, 0.72H, CONHN−*CH*), 7.66 – 7.45 (m, 3H), 7.27 (t, *J* = 8.3 Hz, 1H), 6.96 – 6.78 (m, 3H), 5.07 (for *Z* isomer, s, 1.49H), 4.72 (d, *J* = 4.3 Hz, 2H), 4.65 (for *E* isomer, s, 0.47H), 2.24 (s, 3H); ^13^C NMR (101 MHz, DMSO-*d_6_*) δ 168.6, 164.9, 164.0, 144.9, 143.1, 137.1, 137.0, 133.5, 131.4, 131.3, 127.1, 124.1, 123.5, 117.4, 117.3, 117.1, 115.7, 113.4, 113.2, 67.5, 42.9, 20.7; ^19^F NMR (377 MHz, DMSO) δ -112.79; HRMS (ESI, m/z): Mass calcd. for C_18_H_16_FN_3_O_3_ [M+Na]^+^ 364.1068, found 364.1067.

*(Z)-N'-(3-bromobenzylidene)-2-(7-methyl-3-oxo-2,3-dihydro-4H-benzo[b][1,4]oxazin-4-yl)acetohydrazide (****5l****)*

White solid; 38% yield over 4 steps, m.p. 280-281 ^o^C; *Z*/*E* = 3:1, ^1^H NMR (400 MHz, DMSO-*d_6_*) δ 11.82 (s, 1H, -*NH*), 8.20 (for *E* isomer, s, 0.24H, CONHN−*CH*), 8.01 (for *Z* isomer, s, 0.70H, CONHN−*CH*), 7.94 (d, *J* = 31.9 Hz, 1H), 7.73 (d, *J* = 7.5 Hz, 1H), 7.62 (d, *J* = 8.0 Hz, 1H), 7.41 (t, *J* = 7.8 Hz, 1H), 6.96 – 6.78 (m, 3H), 5.07 (for *Z* isomer, s, 1.49H), 4.72 (d, *J* = 4.3 Hz, 2H), 4.65 (for *E* isomer, s, 0.57H), 2.24 (s, 3H); ^13^C NMR (101 MHz, DMSO-*d_6_*) δ 168.6, 164.9, 144.9, 142.8, 136.9, 133.5, 133.0, 131.4, 129.5, 127.1, 126.8, 123.5, 122.7, 117.4, 115.8, 67.5, 42.9, 20.7; HRMS (ESI, m/z): Mass calcd. for C_18_H_16_BrN_3_O_3_ [M+Na]^+^ 424.0267, found 424.0265.

*(Z)-N'-(2-fluorobenzylidene)-2-(7-methyl-3-oxo-2,3-dihydro-4H-benzo[b][1,4]oxazin-4-yl)acetohydrazide (****5m****)*

White solid; 42% yield over 4 steps, m.p. 210-211 ^o^C; *Z*/*E* = 3:1, ^1^H NMR (400 MHz, DMSO-*d_6_*) δ 11.83 (s, 1H, -*NH*), 8.46 (for *E* isomer, s, 0.23H, CONHN−*CH*), 8.25 (for *Z* isomer, s, 0.79H, CONHN−*CH*), 8.03 – 7.84 (m, 1H), 7.53 – 7.45 (m, 1H), 7.30 (q, *J* = 8.0, 7.5 Hz, 2H), 6.95 – 6.79 (m, 3H), 5.06 (for *Z* isomer, s, 1.54H), 4.72 (d, *J* = 4.3 Hz, 2H), 4.64 (for *E* isomer, s, 0.50H), 2.24 (s, 3H); ^13^C NMR (101 MHz, DMSO-*d_6_*) δ 168.5, 164.9, 163.9, 162.4, 159.9, 144.9, 137.3, 133.7, 133.5, 132.4, 127.1, 127.0, 126.8, 125.4, 123.5, 122.1, 122.0, 117.4, 116.6, 116.4, 115.7, 115.6, 67.5, 42.8, 20.7; ^19^F NMR (377 MHz, DMSO) δ -121.18; HRMS (ESI, m/z): Mass calcd. for C_18_H_16_FN_3_O_3_ [M+Na]^+^ 364.1068, found 364.1066.

*(Z)-2-(7-methyl-3-oxo-2,3-dihydro-4H-benzo[b][1,4]oxazin-4-yl)-N'-propylideneacetohydrazide (****5n****)*

White solid; 37% yield over 4 steps, m.p. 201-202 ^o^C; *Z*/*E* = 3:1, ^1^H NMR (400 MHz, DMSO-*d_6_*) δ 11.26 (s, 1H, -*NH*), 7.53 (for *E* isomer, t, *J* = 8.0 Hz, 0.30H, CONHN−*CH*), 7.38 (for *Z* isomer, t, *J* = 4.9 Hz, 0.74H, CONHN−*CH*), 6.82 (d, *J* = 15.5 Hz, 3H), 4.85 (for *Z* isomer, s, 1.50H), 4.65 (d, *J* = 5.6 Hz, 2H), 4.52 (for *E* isomer, s, 0.56H), 2.30 – 2.24 (m, 2H), 2.23 (d, *J* = 1.7 Hz, 3H), 1.05 (q, *J* = 7.3 Hz, 3H); ^13^C NMR (101 MHz, DMSO-*d_6_*) δ 167.7, 164.8, 164.7, 163.2, 152.9, 149.9, 133.6, 133.4, 127.1, 127.0, 123.5, 123.4, 117.4, 115.6, 67.4, 42.6, 25.7, 20.7, 10.8; HRMS (ESI, m/z): Mass calcd. for C_14_H_17_N_3_O_3_ [M+Na]^+^ 298.1162, found 298.1160.

*(Z)-N'-benzylidene-2-(6-chloro-3-oxo-2,3-dihydro-4H-benzo[b][1,4]oxazin-4-yl)acetohydrazide (****5o****)*

White solid; 47% yield over 4 steps, m.p. 268-270 ^o^C; *Z*/*E* = 3:1, ^1^H NMR (400 MHz, DMSO-*d_6_*) δ 11.73 (s, 1H, -*NH*), 8.24 (for *E* isomer, s, 0.24H, CONHN−*CH*), 8.08 (for *Z* isomer, s, 0.74H, CONHN−*CH*), 7.80 – 7.68 (m, 2H), 7.51 – 7.41 (m, 3H), 7.20 (d, *J* = 7.5 Hz, 1H), 7.06 (d, *J* = 3.4 Hz, 2H), 5.12 (for *Z* isomer, s, 1.48H), 4.74 (s, 2H), 4.70 (for *E* isomer, s, 0.44H); ^13^C NMR (101 MHz, DMSO-*d_6_*) δ 168.3, 164.9, 163.7, 147.6, 144.5, 144.0, 134.5, 131.0, 130.7, 130.5, 129.3, 127.6, 127.5, 126.9, 123.6, 123.5, 118.4, 116.0, 67.4, 43.1; HRMS (ESI, m/z): Mass calcd. for C_17_H_14_ClN_3_O_3_ [M+Na]^+^ 366.0616, found 366.0611.

*(Z)-2-(6-chloro-3-oxo-2,3-dihydro-4H-benzo[b][1,4]oxazin-4-yl)-N'-(4-fluorobenzylidene)acetohydrazide (****5p****)*

White solid; 44% yield over 4 steps, m.p. 282-284 ^o^C; *Z*/*E* = 3:1, ^1^H NMR (400 MHz, DMSO-*d_6_*) δ 11.73 (s, 1H, -*NH*), 8.24 (for *E* isomer, s, 0.24H, CONHN−*CH*), 8.05 (for *Z* isomer, s, 0.77H, CONHN−*CH*), 7.86 – 7.74 (m, 2H), 7.30 (t, *J* = 8.8 Hz, 2H), 7.20 (d, *J* = 9.6 Hz, 1H), 7.06 (s, 2H), 5.12 (for *Z* isomer, s, 1.49H), 4.74 (s, 2H), 4.70 (for *E* isomer, s, 0.50H); ^13^C NMR (101 MHz, DMSO-*d_6_*) δ 168.3, 164.9, 164.8, 163.7, 162.3, 146.5, 144.0, 143.4, 131.1, 131.0, 129.8, 129.7, 129.6, 126.9, 123.6, 123.5, 118.4, 116.5, 116.3, 116.2, 116.0, 67.4, 43.1; ^19^F NMR (377 MHz, DMSO) δ -110.41, -110.65; HRMS (ESI, m/z): Mass calcd. for C_17_H_13_ClFN_3_O_3_ [M+Na]^+^ 384.0522, found 384.0517.

*(Z)-2-(6-chloro-3-oxo-2,3-dihydro-4H-benzo[b][1,4]oxazin-4-yl)-N'-(3-fluorobenzylidene)acetohydrazide (****5q****)*

White solid; 42% yield over 4 steps, m.p. 264-266 ^o^C; *Z*/*E* = 3:1, ^1^H NMR (400 MHz, DMSO-*d_6_*) δ 11.83 (s, 1H, -*NH*), 8.25 (for *E* isomer, s, 0.22H, CONHN−*CH*), 8.05 (for *Z* isomer, s, 0.78H, CONHN−*CH*), 7.65 – 7.47 (m, 3H), 7.27 (t, *J* = 9.1 Hz, 1H), 7.20 (d, *J* = 8.8 Hz, 1H), 7.06 (d, *J* = 4.3 Hz, 2H), 5.14 (for *Z* isomer, s, 1.53H), 4.74 (s, 2H), 4.71 (for *E* isomer, s, 0.44H); ^13^C NMR (101 MHz, DMSO-*d_6_*) δ 168.5, 164.9, 164.1, 163.9, 161.7, 144.0, 143.1, 137.1, 137.0, 131.4, 131.3, 131.0, 126.9, 124.1, 123.6, 123.5, 118.4, 117.3, 117.1, 116.0, 113.4, 113.2, 67.4, 43.1; ^19^F NMR (377 MHz, DMSO) δ -112.80; HRMS (ESI, m/z): Mass calcd. for C_17_H_13_ClFN_3_O_3_ [M+Na]^+^ 384.0522, found 384.0519.

*(Z)-2-(6-chloro-3-oxo-2,3-dihydro-4H-benzo[b][1,4]oxazin-4-yl)-N'-(2-fluorobenzylidene)acetohydrazide (****5r****)*

White solid; 41% yield over 4 steps, m.p. 220-222 ^o^C; *Z*/*E* = 3:1, ^1^H NMR (400 MHz, DMSO-*d_6_*) δ 11.84 (s, 1H, -*NH*), 8.46 (for *E* isomer, s, 0.22H, CONHN−*CH*), 8.26 (for *Z* isomer, s, 0.77H, CONHN−*CH*), 7.93 (dt, *J* = 48.0, 6.8 Hz, 1H), 7.50 (q, *J* = 7.2 Hz, 1H), 7.35 – 7.26 (m, 2H), 7.20 (s, 1H), 7.06 (d, *J* = 3.8 Hz, 2H), 5.12 (for *Z* isomer, s, 1.54H), 4.74 (s, 2H), 4.70 (for *E* isomer, s, 0.43H); ^13^C NMR (101 MHz, DMSO-*d_6_*) δ 168.5, 165.0, 163.8, 162.4, 159.9, 144.0, 137.3, 137.3, 132.4, 131.0, 127.0, 125.4, 123.5, 122.1, 122.0, 118.4, 116.6, 116.4, 116.0, 67.4, 43.1; ^19^F NMR (376 MHz, DMSO) δ -120.98, -121.21; HRMS (ESI, m/z): Mass calcd. for C_17_H_13_ClFN_3_O_3_ [M+Na]^+^ 384.0522, found 384.0518.

*(Z)-2-(6-chloro-3-oxo-2,3-dihydro-4H-benzo[b][1,4]oxazin-4-yl)-N'-propylideneacetohydrazide (****5s****)*

White solid; 36% yield over 4 steps, m.p. 206-208 ^o^C; *Z*/*E* = 3:1, ^1^H NMR (400 MHz, DMSO-*d_6_*) δ 11.29 (s, 1H, -*NH*), 7.54 (for *E* isomer, t, *J* = 15 Hz, 0.30H, CONHN−*CH*), 7.39 (for *Z* isomer, t, *J* = 7.2 Hz, 0.74H, CONHN−*CH*), 7.11 (d, *J* = 19.4 Hz, 1H), 7.06 – 7.03 (m, 2H), 4.92 (for *Z* isomer, s, 1.46H), 4.74 (s, 2H), 4.58 (for *E* isomer, s, 0.56H), 2.30 – 2.21 (m, 2H), 1.06 (t, *J* = 7.6 Hz, 3H); ^13^C NMR (101 MHz, DMSO-*d_6_*) δ 167.6, 164.9, 163.1, 152.9, 150.0, 144.0, 131.0, 126.9, 123.5, 123.4, 118.4, 118.3, 115.9, 115.9, 67.3, 42.8, 25.7, 10.8; ^19^F NMR (376 MHz, DMSO) δ -120.98, -121.21; HRMS (ESI, m/z): Mass calcd. for C_13_H_14_ClFN_3_O_3_ [M+Na]^+^ 318.0616, found 318.0615.

**^1^H NMR, ^13^C NMR and ^19^F NMR spectra and HRMS of products**

**Figure S1**. ^1^H NMR for compound **5a**

**Figure S2**. ^13^C NMR for compound **5a**


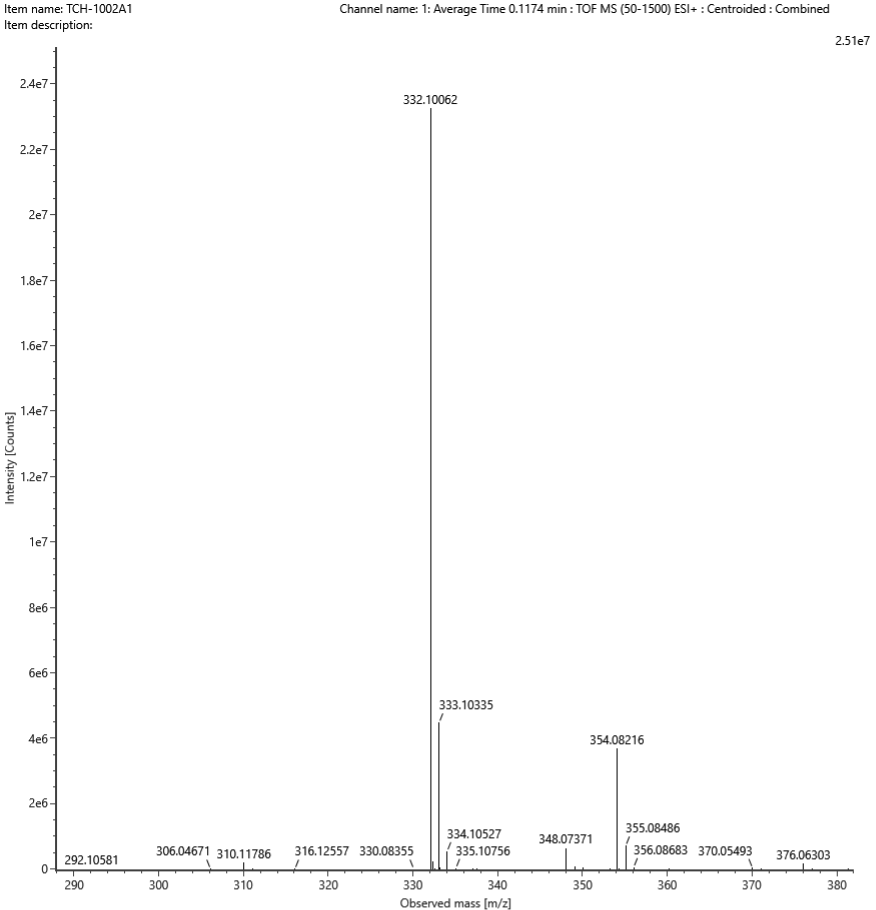


**Figure S3**. HRMS for compound **5a**

**Figure S4**. ^1^H NMR for compound **5b**

**Figure S5**. ^13^C NMR for compound **5b**

**Figure S6**. ^19^F NMR for compound **5b**


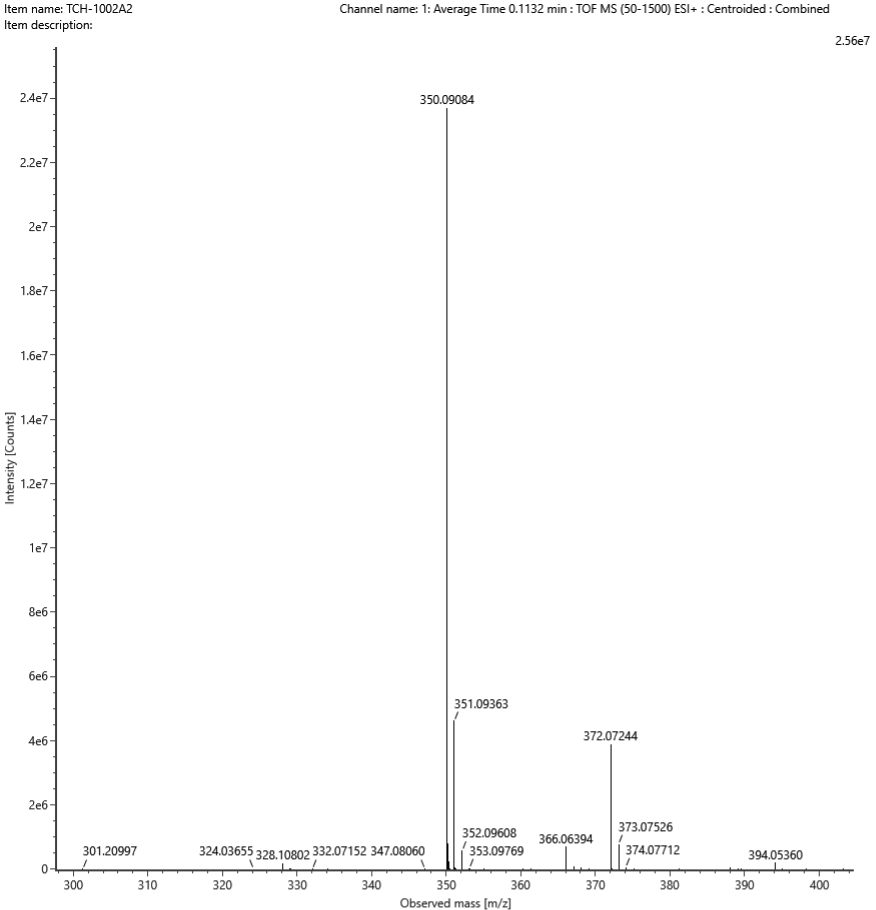


**Figure S7**. HRMS for compound **5b**

**Figure S8**. ^1^H NMR for compound **5c**


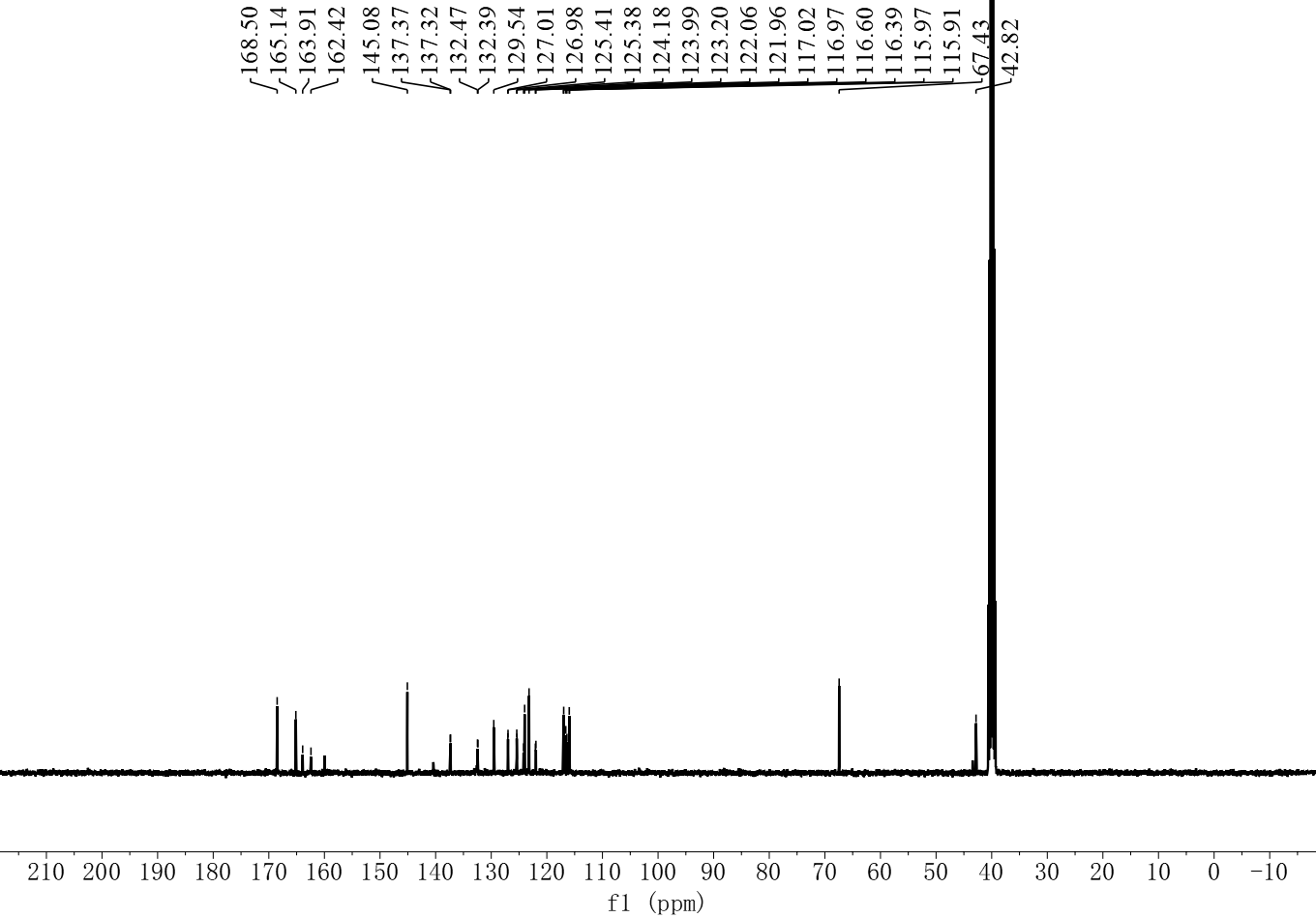


**Figure S9**. ^13^C NMR for compound **5c**

**Figure S10**. ^19^F NMR for compound **5c**


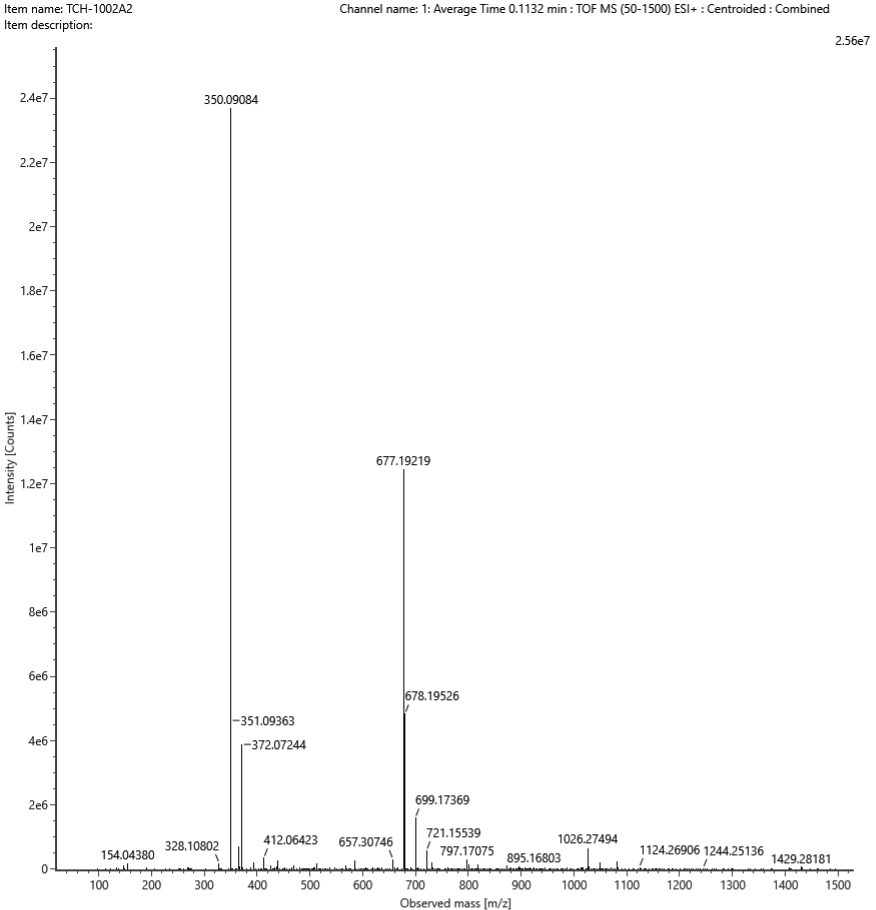


**Figure S11**. HRMS for compound **5c**

**Figure S12**. ^1^H NMR for compound **5d**

**Figure S13**. ^13^C NMR for compound **5d**

**Figure S 14**. ^19^F NMR for compound **5d**


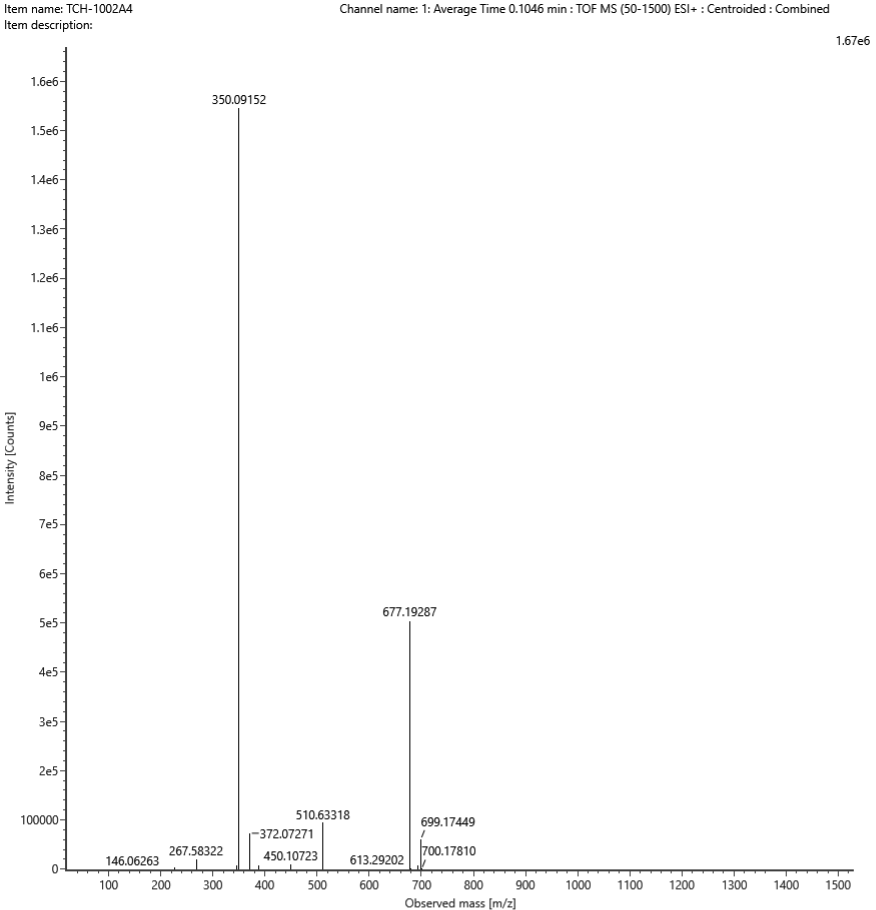


**Figure S15**. HRMS for compound **5d**

**Figure S16**. ^1^H NMR for compound **5e**

**Figure S17**. ^13^C NMR for compound **5e**


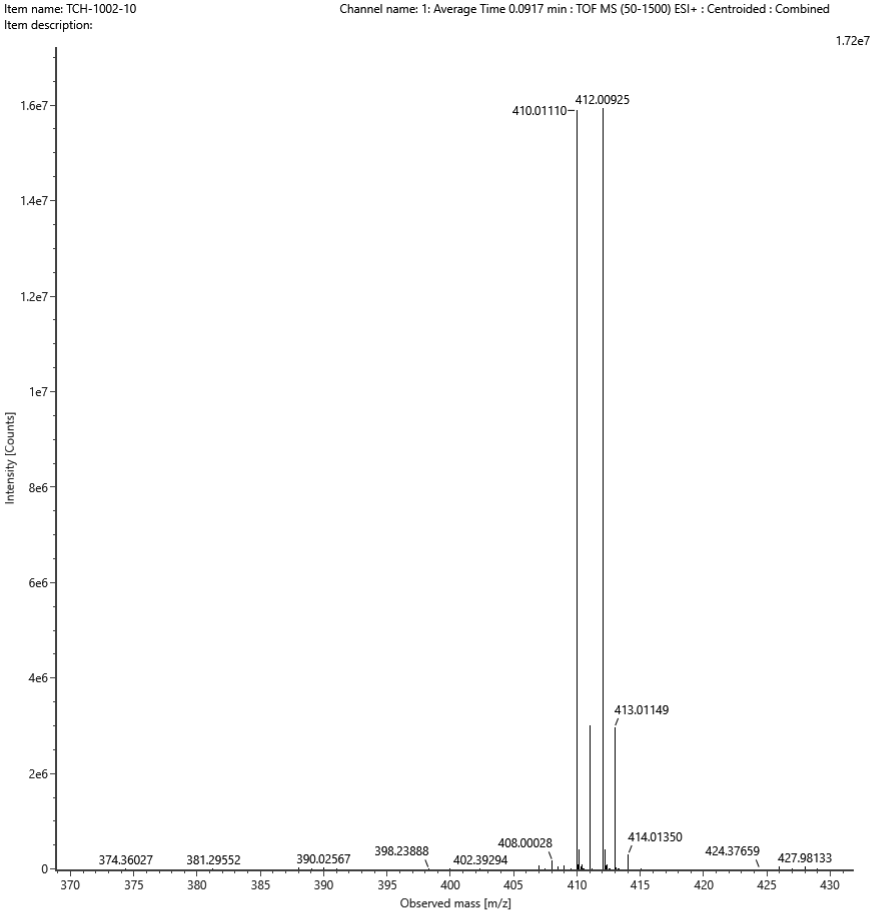


**Figure S18**. HRMS for compound **5e**

**Figure S19**. ^1^H NMR for compound **5f**

**Figure S20**. ^13^C NMR for compound **5f**


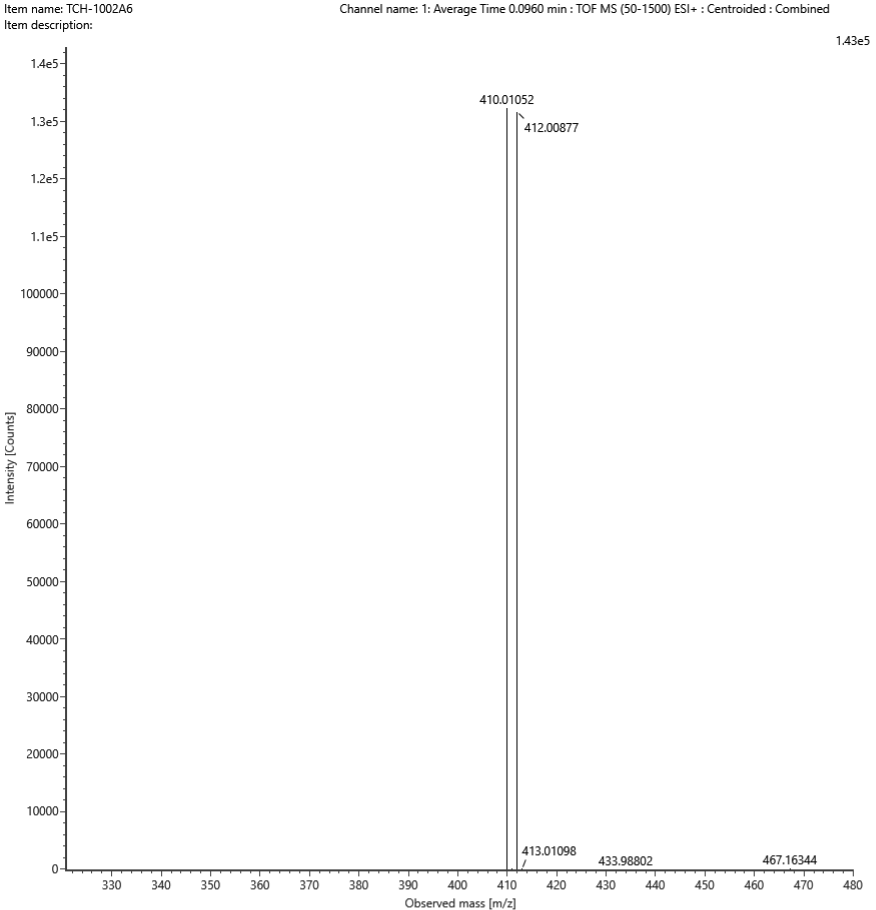


**Figure S21**. HRMS for compound **5f**

**Figure S22**. ^1^H NMR for compound **5g**

**Figure S23**. ^13^C NMR for compound **5g**


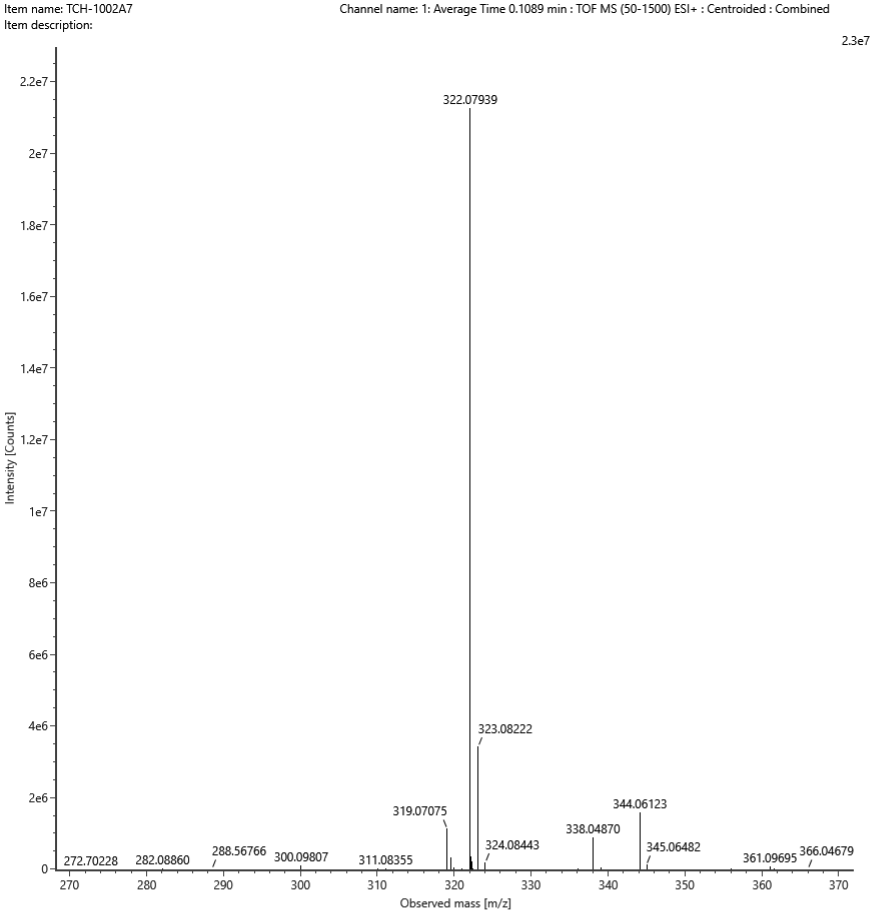


**Figure S24**. HRMS for compound **5g**

**Figure S25**. ^1^H NMR for compound **5h**

**Figure S26**. ^13^C NMR for compound **5h**


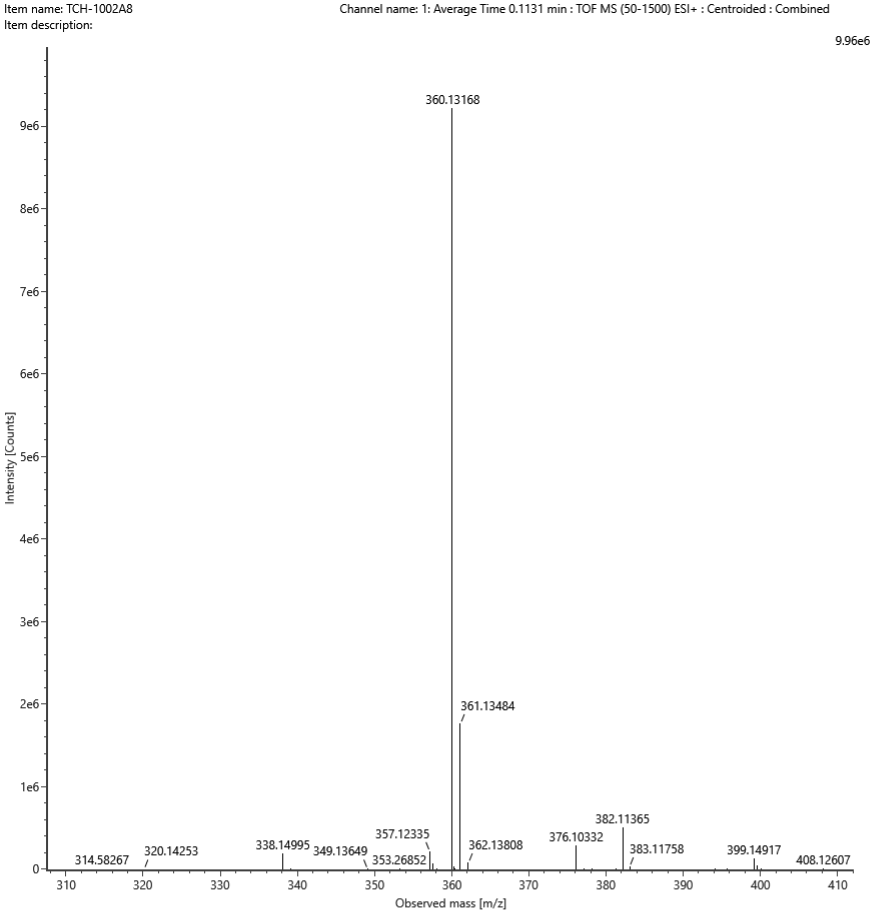


**Figure S27**. HRMS for compound **5h**

**Figure S28**. ^1^H NMR for compound **5i**

**Figure S29**. ^13^C NMR for compound **5i**


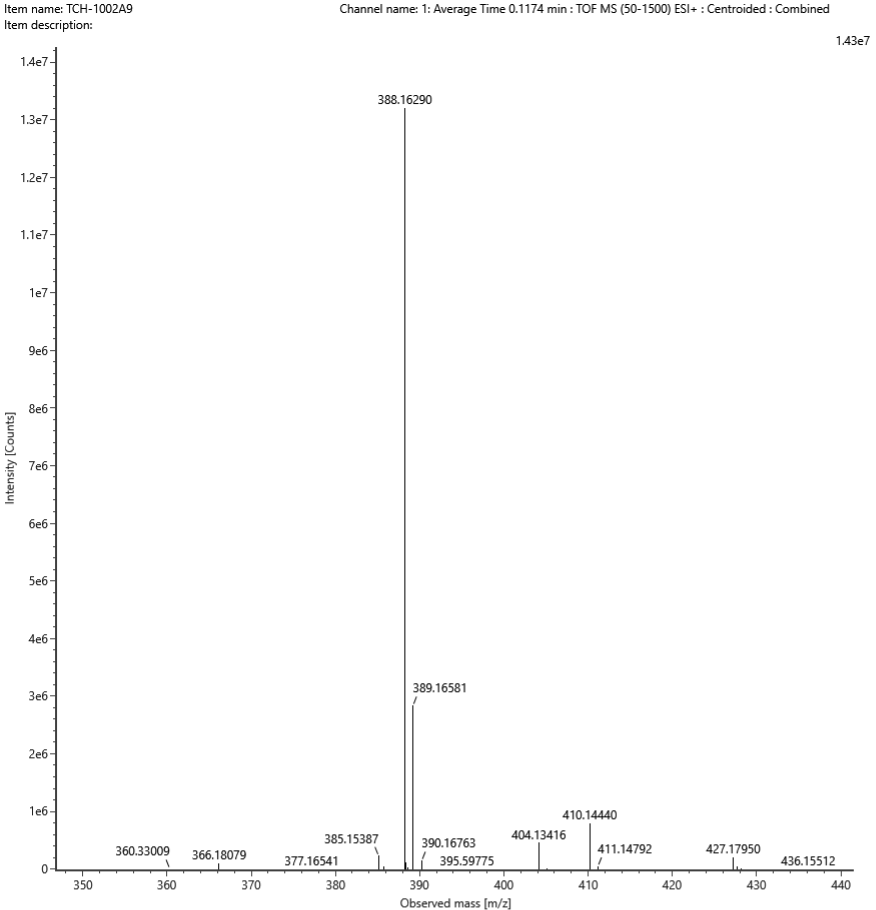


**Figure S30**. HRMS for compound **5i**

**Figure S31**. ^1^H NMR for compound **5j**

**Figure S32**. ^13^C NMR for compound **5j**

**Figure S33**. ^19^F NMR for compound **5j**


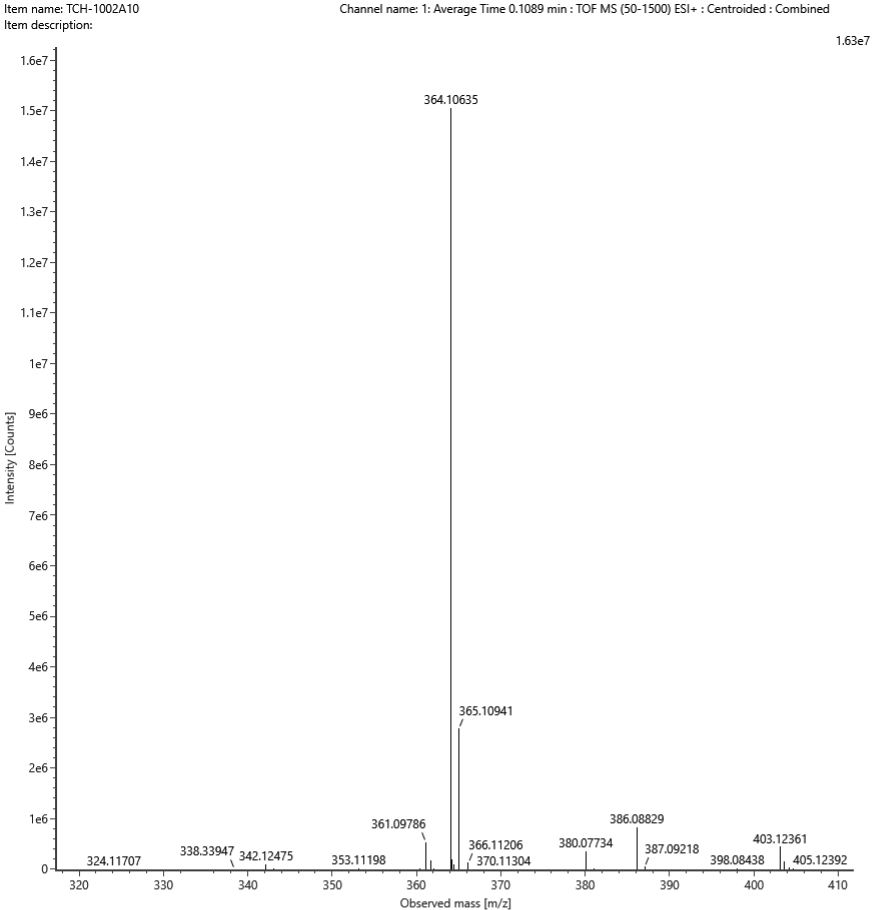


**Figure S34**. HRMS for compound **5j**

**Figure S35**. ^1^H NMR for compound **5k**

**Figure S36**. ^13^C NMR for compound **5k**

**Figure S37**. ^19^F NMR for compound **5k**


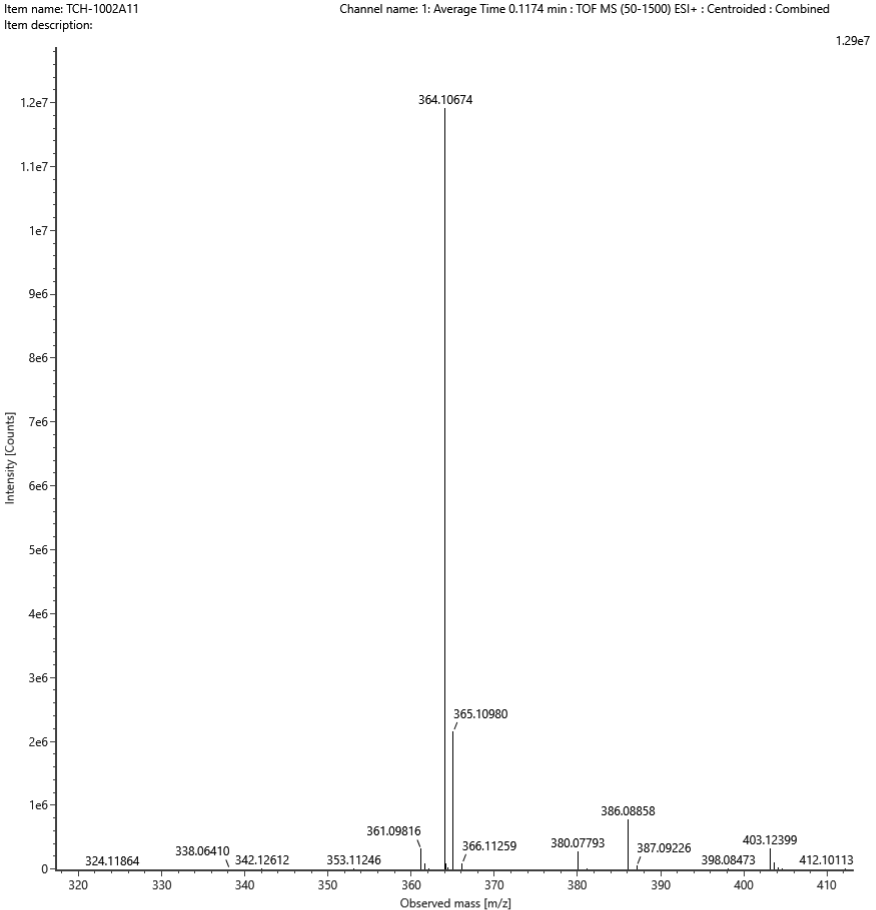


**Figure S38**. HRMS for compound **5k**

**Figure S39**. ^1^H NMR for compound **5l**

**Figure S40**. ^13^C NMR for compound **5l**


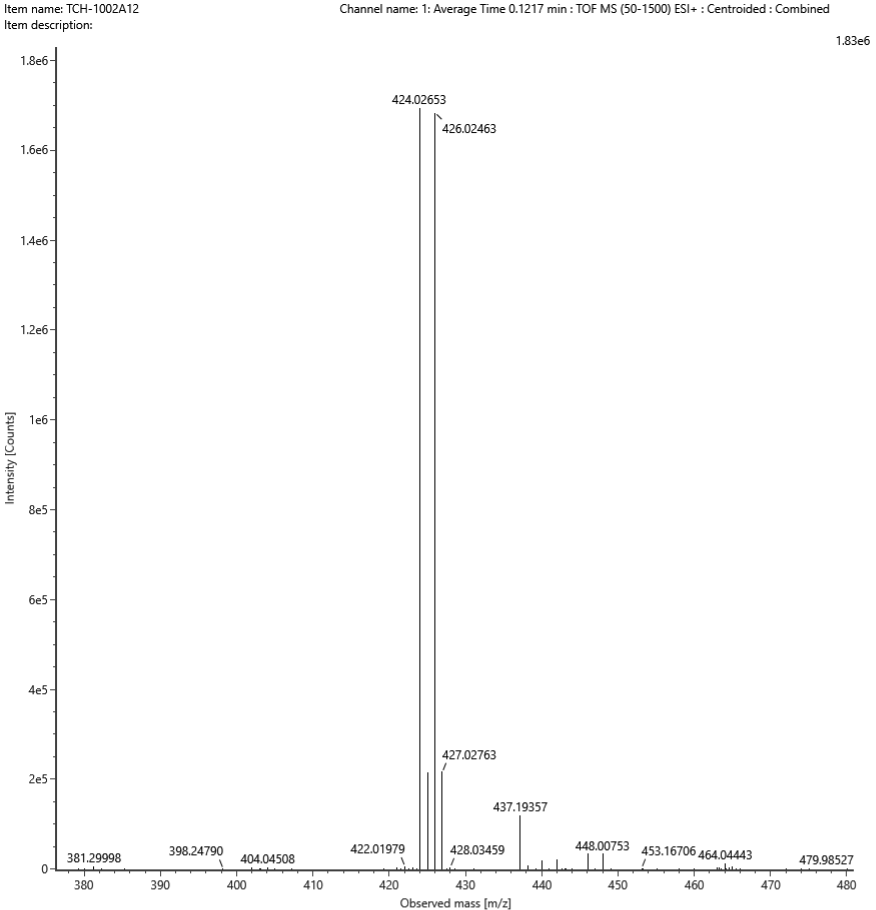


**Figure S41**. HRMS for compound **5l**

**Figure S42**. ^1^H NMR for compound **5m**

**Figure S43**. ^13^C NMR for compound **5m**

**Figure S44**. ^19^F NMR for compound **5m**


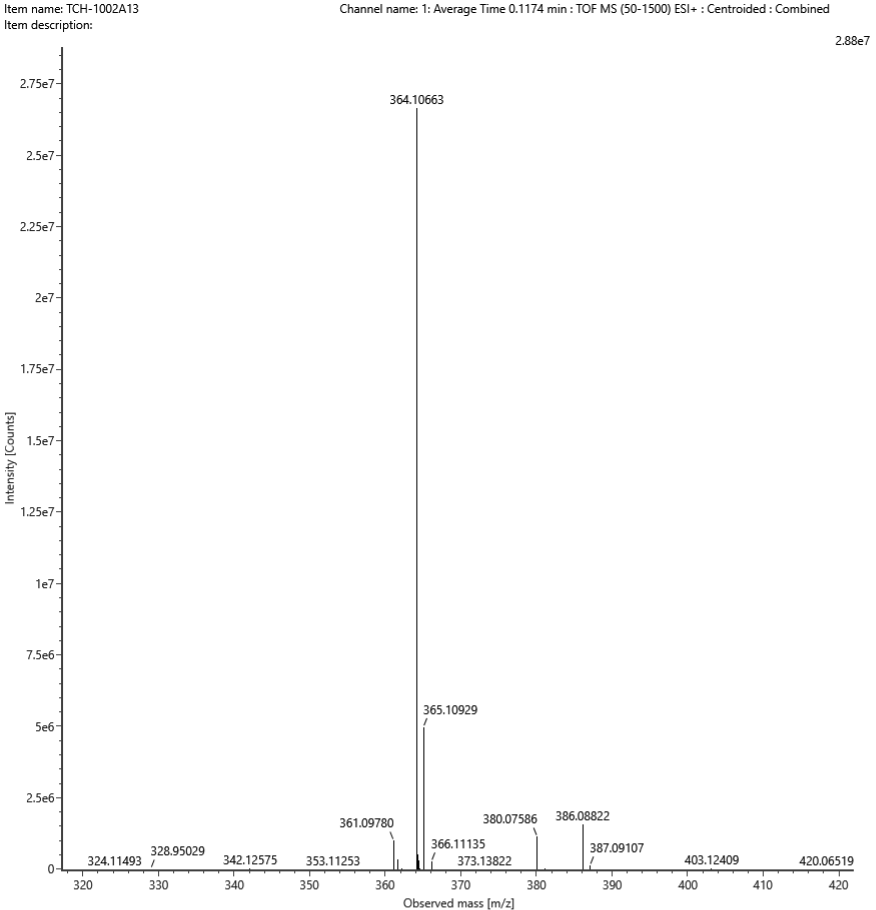


**Figure S45**. HRMS for compound **5m**

**Figure S46**. ^1^H NMR for compound **5n**

**Figure S47**. ^13^C NMR for compound **5n**


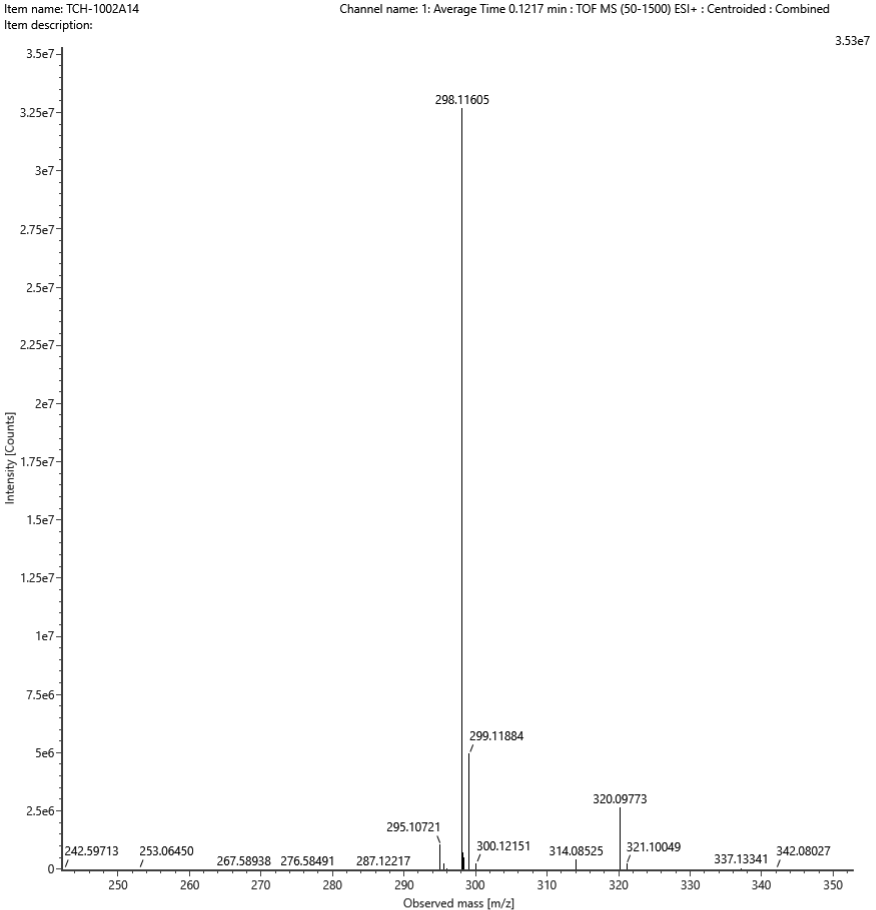


**Figure S48**. HRMS for compound **5n**

**Figure S49**. ^1^H NMR for compound **5o**

**Figure S50**. ^13^C NMR for compound **5o**


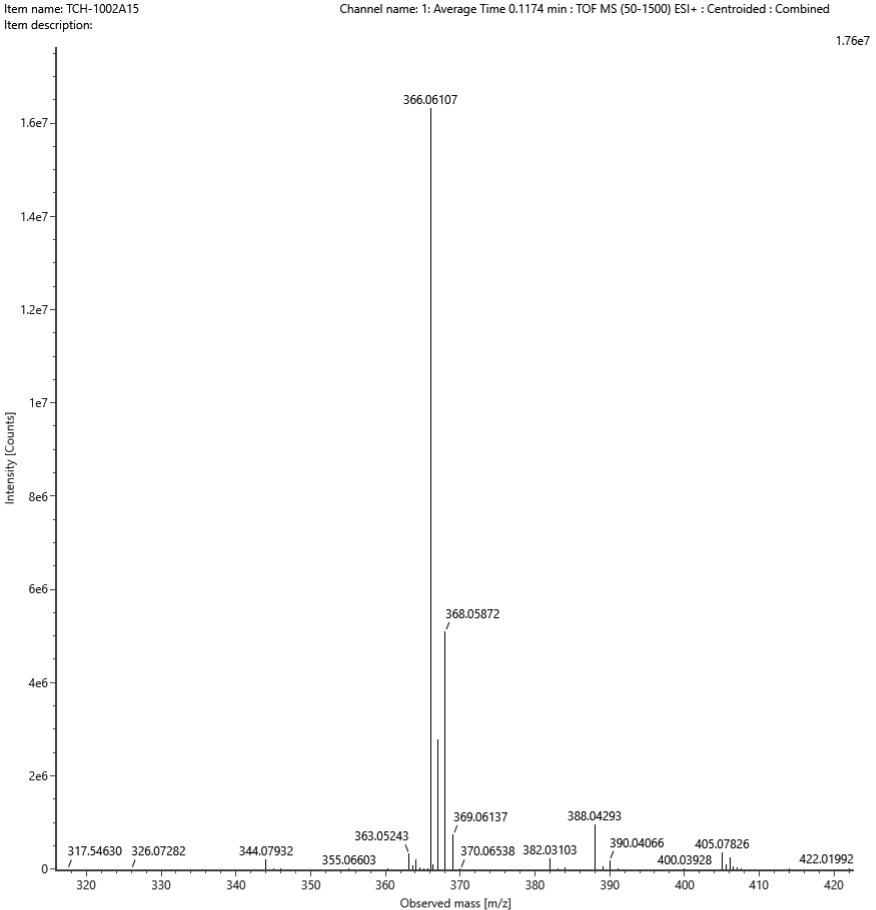


**Figure S51**. HRMS for compound **5o**

**Figure S52**. ^1^H NMR for compound **5p**


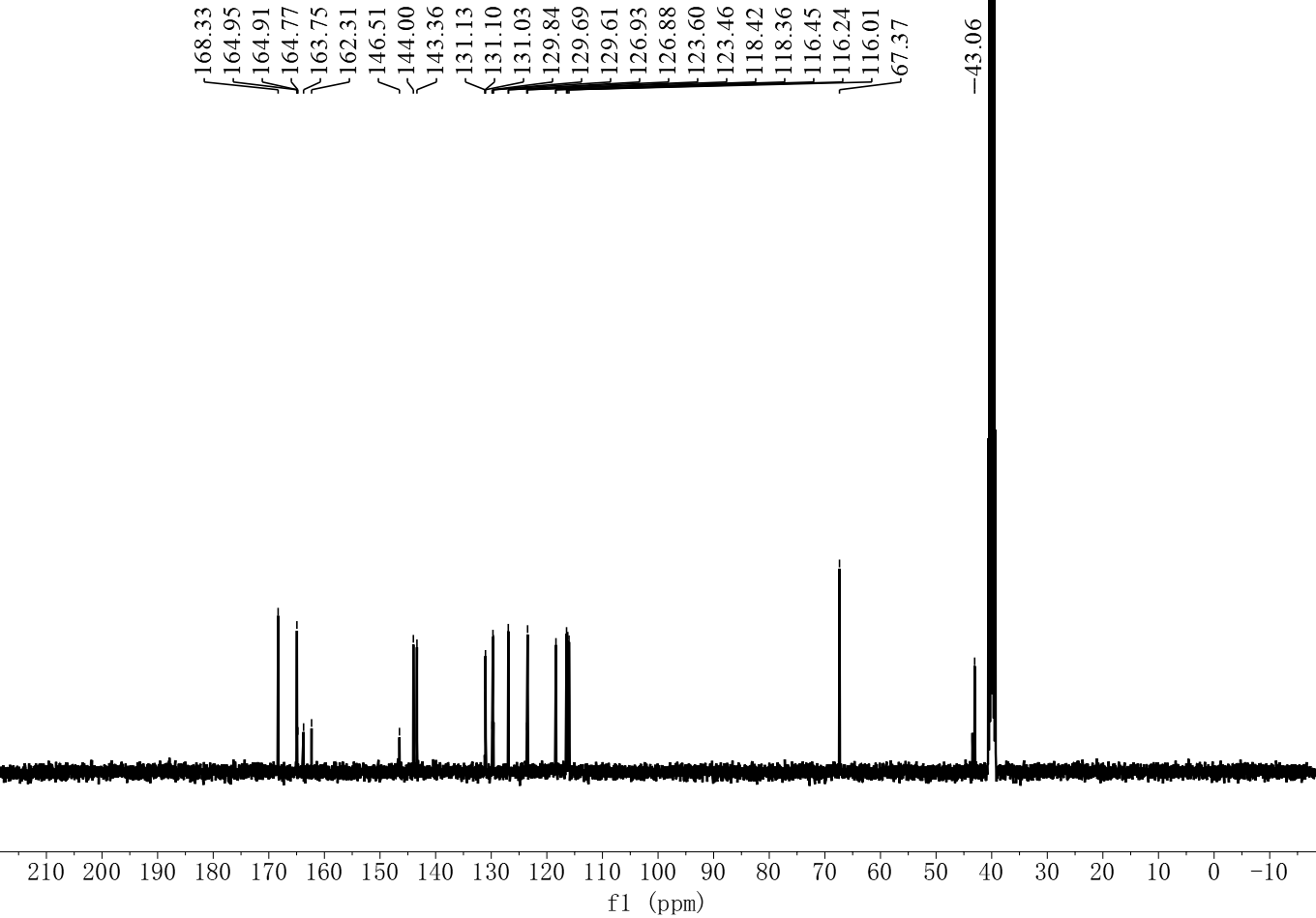


**Figure S53**. ^13^C NMR for compound **5p**


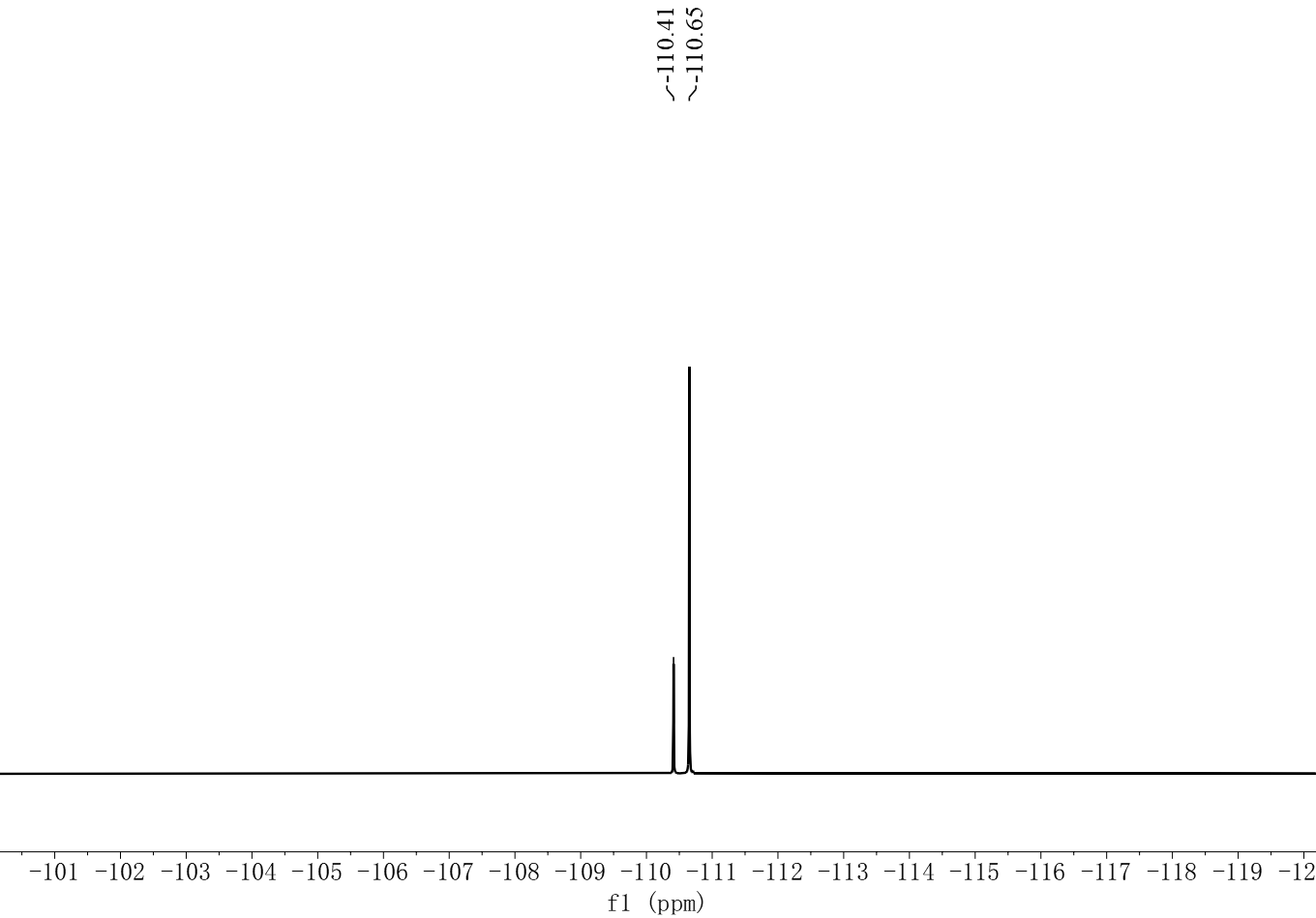


**Figure S54**. ^19^F NMR for compound **5p**


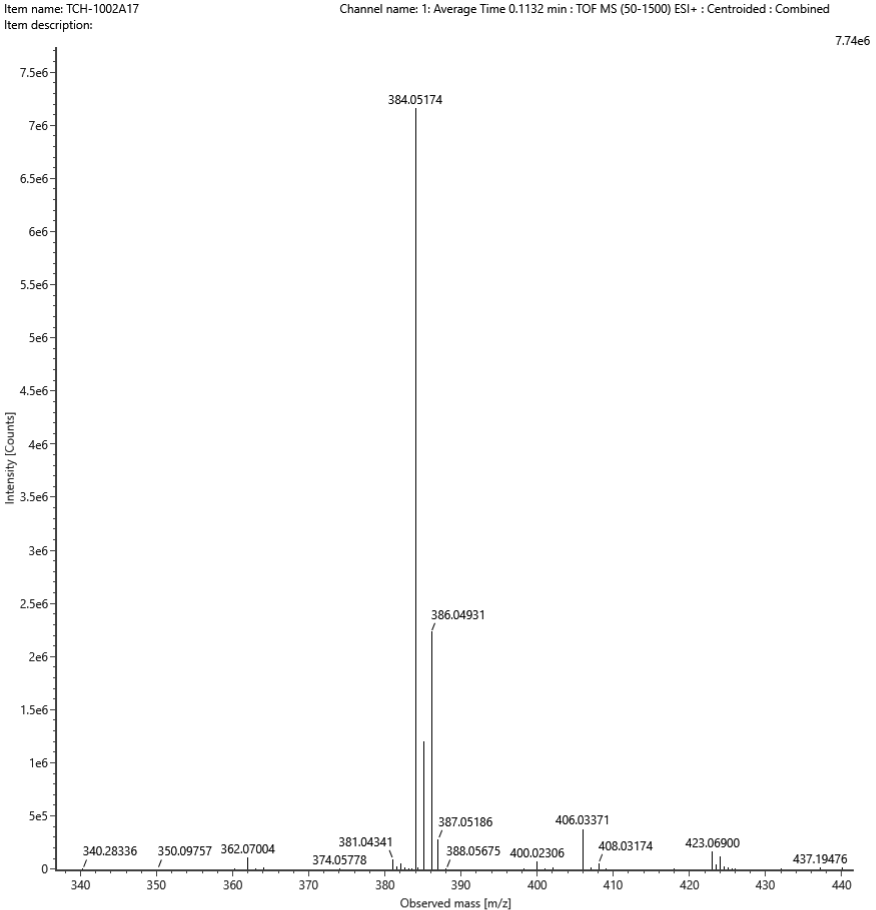


**Figure S55**. HRMS for compound **5p**

**Figure S56**. ^1^H NMR for compound **5q**

**Figure S57**. ^13^C NMR for compound **5q**

**Figure S58**. ^19^F NMR for compound **5q**


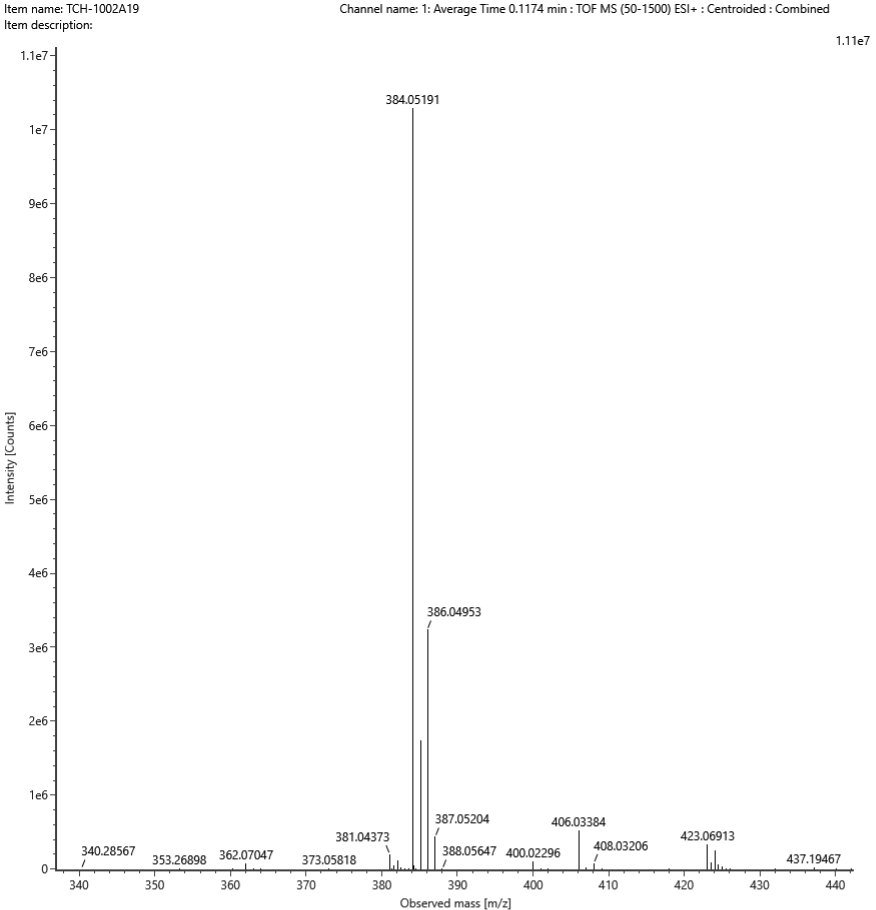


**Figure S59**. HRMS for compound **5q**

**Figure S60**. ^1^H NMR for compound **5r**

**Figure S61**. ^13^C NMR for compound **5r**

**Figure S62**. ^19^F NMR for compound **5r**


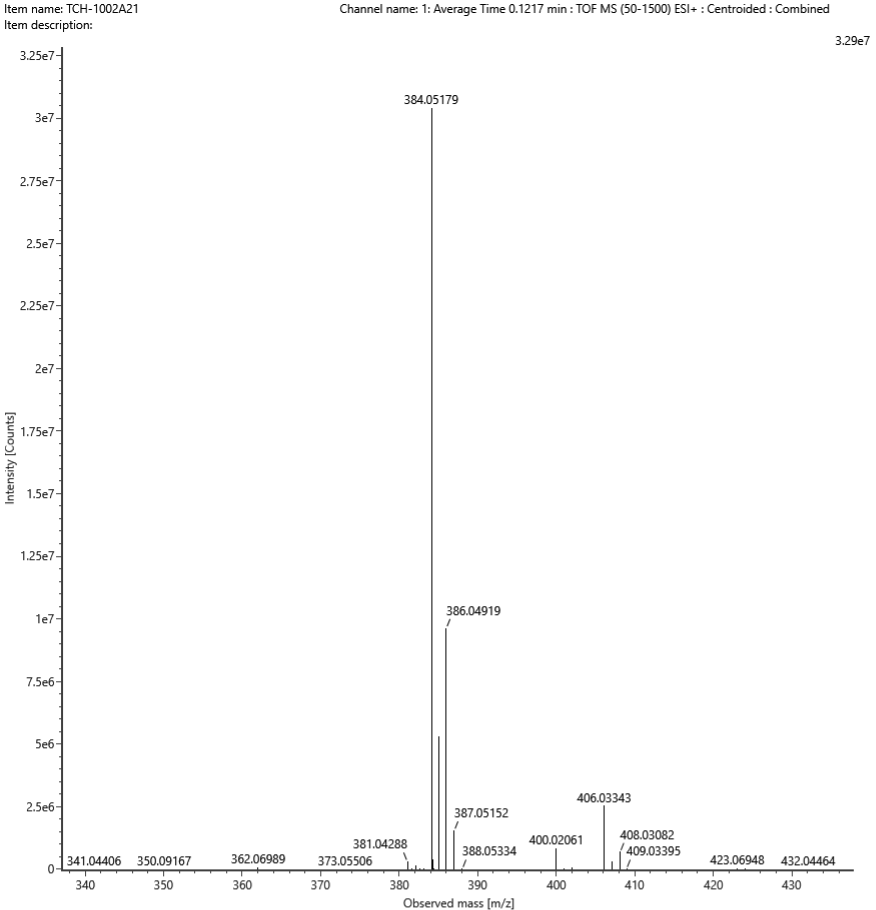


**Figure S63**. HRMS for compound **5r**

**Figure S64**. ^1^H NMR for compound **5s**

**Figure S65**. ^13^C NMR for compound **5s**


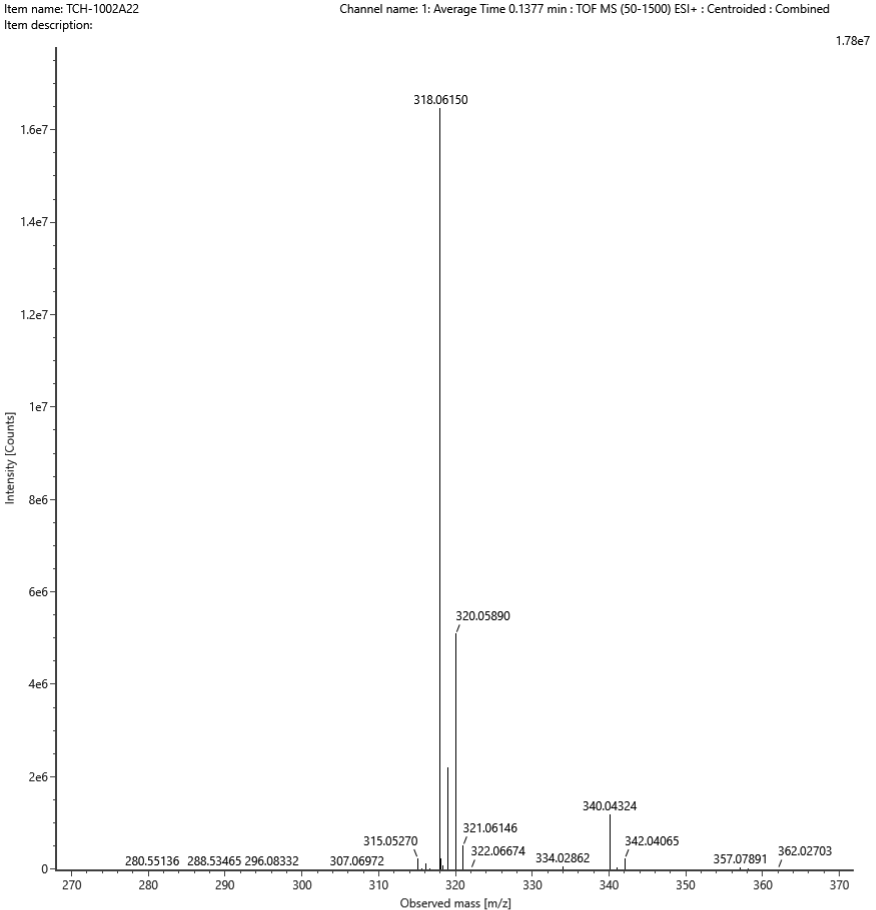


**Figure S66**. HRMS for compound **5s**

Table S1 *In vitro* fungicidal activity of target compounds against tested fungi

| Comp. | Mycelium growth inhibitory rate (%) at 50 *μ*g/mL | | | | |
| --- | --- | --- | --- | --- | --- |
|  | *G. zeae* | *P. sasakii* | *P. infestans* | *C. wilt* | *P. capsici* |
| **5a** | 36.84 ± 5.75 | 43.44 ± 2.49 | 44.76 ± 3.58 | 46.39 ± 1.91 | 24.25 ± 1.68 |
| **5b** | 13.22 ± 2.07 | 44.89 ± 2.31 | 34.76 ± 3.58 | 54.78 ± 2.28 | 15.41 ± 1.28 |
| **5c** | 24.68 ± 1.92 | 44.16 ± 2.69 | 33.10 ± 4.56 | 43.59 ± 1.44 | 17.56 ± 1.50 |
| **5d** | 20.01 ± 3.07 | 45.13 ± 3.03 | 47.86 ± 1.97 | 24.01 ± 1.91 | 25.45 ± 2.03 |
| **5e** | 19.77 ± 1.64 | 42.24 ± 2.58 | 61.90 ± 1.73 | 19.11 ± 1.38 | 23.30 ± 2.68 |
| **5f** | 19.30 ± 2.91 | 43.92 ± 1.69 | 33.81 ± 1.73 | 22.14 ± 1.02 | 32.38 ± 1.68 |
| **5g** | 35.91 ± 4.13 | 45.37 ± 3.35 | 37.38 ± 2.29 | 25.64 ± 2.24 | 29.51 ± 2.11 |
| **5h** | 31.23 ± 3.32 | 41.28 ± 2.98 | 55.48 ± 3.55 | 45.69 ± 2.41 | 27.60 ± 1.50 |
| **5i** | 30.99 ± 3.00 | 39.83 ± 1.75 | 43.10 ± 3.31 | 41.03 ± 2.06 | 31.42 ± 1.68 |
| **5j** | 50.18 ± 4.92 | 50.66 ± 1.09 | 41.19 ± 1.08 | 52.91 ± 1.69 | 34.05 ± 2.03 |
| **5k** | 30.29 ± 4.13 | 46.33 ± 1.42 | 37.14 ± 2.02 | 40.09 ± 2.57 | 27.36 ± 2.51 |
| **5l** | 76.37 ± 2.07 | 64.38 ± 4.35 | 38.33 ± 2.77 | 43.59 ± 2.45 | 18.28 ± 2.03 |
| **5m** | 37.78 ± 4.04 | 43.20 ± 4.15 | 32.14 ± 2.35 | 38.23 ± 1.05 | 16.13 ± 1.50 |
| **5n** | 37.31 ± 4.50 | 44.89 ± 3.47 | 39.52 ± 4.30 | 41.96 ± 2.30 | 30.47 ± 1.50 |
| **5o** | 76.14 ± 1.26 | 54.27 ± 1.75 | 32.14 ± 1.97 | 40.33 ± 1.44 | 33.33 ± 1.50 |
| **5p** | 33.10 ± 6.86 | 44.40 ± 3.26 | 33.57 ± 2.17 | 71.33 ± 1.47 | 32.86 ± 2.11 |
| **5q** | 51.11 ± 3.81 | 73.32 ± 1.09 | 43.10 ± 1.40 | 39.16 ± 1.47 | 32.14 ± 1.48 |
| **5r** | 41.52 ± 2.61 | 57.40 ± 1.99 | 52.38 ± 2.66 | 45.92 ± 2.60 | 25.93 ± 2.34 |
| **5s** | 39.18 ± 3.03 | 44.16 ± 4.63 | 82.62 ± 1.08 | 48.02 ± 2.06 | 15.17 ± 2.11 |
| **5t** | 34.50 ± 2.29 | 38.15 ± 3.81 | 34.29 ± 2.02 | 43.59 ± 1.44 | 13.74 ± 1.68 |
| Hymexazol | 49.47 ± 1.26 | 60.77 ± 1.69 | 72.86 ± 2.02 | 49.88 ± 1.05 | 27.60 ± 1.50 |
| Carbendazim | 94.85 ± 1.70 | 81.95 ± 1.51 | 58.57 ± 1.28 | 68.30 ± 1.44 | 33.81 ± 2.11 |

Table S2 The EC_50_ values of selected target compounds against tested fungi

| Pathogens | Comp. | Regression equation | EC_50_ (*u*g/mL) | Pathogens | Comp. | Regression equation | EC_50_ (*u*g/mL) |
| --- | --- | --- | --- | --- | --- | --- | --- |
| *G. zeae* | **5l** | y = 1.7566x + 2.7123 | 20.06 ± 0.26 | *P. infestans* | **5e** | y = 1.3509x + 3.0713 | 26.77 ± 0.39 |
|  | **5o** | y = 2.0930x + 2.1434 | 23.17 ± 0.13 |  | **5s** | y = 1.8439x + 2.8121 | 15.37 ± 0.07 |
|  | Hymexazol | y = 1.6843x + 2.2924 | 40.51 ± 1.85 |  | Hymexazol | y = 1.4933x + 3.1129 | 18.35 ± 0.51 |
|  | Carbendazim | y = 1.7550x + 3.4522 | 7.62 ± 0.02 |  | Carbendazim | y = 1.6463x + 2.4701 | 34.41 ± 2.43 |
| *P. sasakii* | **5l** | y = 2.0253x + 1.9925 | 30.55 ± 1.25 | *C. wilt* | **5b** | y = 1.7469x + 1.8574 | > 50 |
|  | **5q** | y = 1.9901x + 2.1623 | 26.66 ± 0.36 |  | **5p** | y = 1.5894x + 2.7575 | 26.76 ± 0.57 |
|  | Hymexazol | y = 2.0632x + 1.8785 | 32.77 ± 1.47 |  | Hymexazol | y = 1.7360x + 1.6936 | > 50 |
|  | Carbendazim | y = 1.8675x + 2.7220 | 16.59 ± 0.42 |  | Carbendazim | y = 1.7876x + 2.4682 | 26.08 ± 1.25 |
